# Nuclear Receptor-Targeted Therapies: Reprogramming Metabolism with TRβ, ERRα, and LXR Modulators

**DOI:** 10.3390/biom16020272

**Published:** 2026-02-09

**Authors:** Carmen Di Giovanni, Antonio Lavecchia

**Affiliations:** “Drug Discovery” Laboratory, Department of Pharmacy, University of Naples Federico II, I-80131 Naples, Italy; carmen.digiovanni@unina.it

**Keywords:** nuclear receptors, metabolic disorders, targeted therapy, thyroid hormone receptor β, estrogen-related receptor α, liver X receptor, small-molecule modulators, non-alcoholic fatty liver disease, mitochondrial biogenesis

## Abstract

Metabolic disorders, including metabolic dysfunction-associated fatty liver disease (MAFLD), obesity, and dyslipidemia, impose a substantial and escalating global health burden, highlighting an urgent need for effective pharmacotherapies. Selective modulation of nuclear receptors (NRs) has emerged as a promising strategy to restore metabolic homeostasis. This review focuses on three therapeutically pivotal yet under-explored NRs: thyroid hormone receptor β (TRβ), estrogen-related receptor α (ERRα), and liver X receptor (LXRα/β). We critically examine recent advances in the development of small-molecule modulators for these targets and discuss their translational potential. TRβ agonists, including resmetirom (MGL-3196) and VK2809, have demonstrated compelling efficacy in clinical trials for metabolic dysfunction-associated steatohepatitis (MASH), significantly reducing hepatic steatosis and fibrosis. Next-generation hepatoselective modulators such as TG68 enhance tissue specificity and potency. ERRα, a master regulator of mitochondrial biogenesis and energy metabolism, is targeted by inverse agonists (compound **29**, GSK5182) and agonists (JND003, SLU-PP-915), which show promise in ameliorating insulin resistance and promoting lipid oxidation in preclinical obesity models. LXRs, central players in cholesterol homeostasis, are the focus of innovative drug design aimed at harnessing atheroprotective benefits via LXRβ-selective or partial agonists, thereby circumventing adverse effects on triglyceride synthesis. Collectively, the ongoing development of TRβ, ERRα, and LXR modulators exemplifies a new frontier in precision medicine, offering powerful approaches to reprogram dysregulated metabolic pathways with substantial promise for treating metabolic diseases.

## 1. Introduction

Metabolic disorders represent a major and escalating global health challenge, contributing significantly to mortality, morbidity, and socioeconomic burden [1]. Table 1 highlights the global mortality burden attributable to key metabolic risk factors.

Obesity, MAFLD, MASH, and atherosclerosis constitute interconnected manifestations of a common pathological spectrum driven by insulin resistance, chronic low-grade inflammation, endothelial dysfunction, and disrupted lipid and glucose metabolism [12]. Obesity, affecting over one billion individuals worldwide, serves as the primary modifiable risk factor, promoting the development of MAFLD, type 2 diabetes (T2D), and cardiovascular disease (CVD) [13,14]. MAFLD, currently the leading cause of chronic liver disease, affects approximately 30% of adults globally and substantially increases risks of cirrhosis, hepatocellular carcinoma (HCC), and cardiovascular complications [15]. Atherosclerosis, the principal cause of myocardial infarction and stroke, remains the leading cause of death worldwide, with rising prevalence in low- and middle-income countries attributable to westernized lifestyles and epidemiological transition. These conditions frequently coexist, amplifying risks of severe outcomes; however, public awareness and early diagnosis remain inadequate. Effective management requires integrated strategies combining nutrition, physical activity, reduced sedentary behavior, food-environment regulation, and the development of novel pharmacological therapies. Recent advances in glucagon-like peptide-1 receptor agonists (GLP-1 RAs) and sodium–glucose cotransporter-2 inhibitors (SGLT2i) have shown promise for obesity and MASH management [16,17].

Nuclear receptors (NRs) constitute a superfamily of ligand-activated transcription factors fundamental to metabolism, development, and physiological homeostasis [18]. NRs can be classified into four principal categories. Type I receptors are steroid hormone receptors that typically remain inactive in the absence of a ligand and migrate to the nucleus upon ligand binding. Type II receptors, commonly known as non-steroid receptors, are usually nuclear and can bind DNA in the presence or absence of ligands, often forming heterodimers with retinoid X receptors (RXRs). Type III receptors, also called orphan receptors, lack identified endogenous ligands yet share structural features with other NRs. Type IV receptors function as monomeric DNA-binding proteins, binding specific hormone response elements (HREs) independently and regulating distinct target gene sets [19]. Each receptor subtype plays a distinct role in controlling essential physiological processes, including metabolic balance, inflammatory responses, endocrine signaling, and cellular differentiation. Mainly, NRs are controlled by endogenous small lipophilic molecules, while others remain “orphan” receptors with no identified physiological ligand. Ligand engagement induces conformational changes enabling DNA binding at specific response elements genome-wide. Upon chromatin binding, NRs recruit coregulators, chromatin-remodeling complexes, and components of the general transcription assembly to activate or repress gene expression [20]. Functioning as molecular sensors, they integrate hormonal, nutritional, and metabolic cues into transcriptional programs governing lipid metabolism, glucose regulation, energy balance, and inflammatory responses. Consequently, NRs are regarded as “master regulators” of metabolism and represent key therapeutic targets for metabolic diseases [21].

Beyond the metabolic NRs discussed in detail in this review, additional NR family members, notably the glucocorticoid receptor (GR) and retinoid X receptors (RXRs), play critical roles in the regulation of lipid and glucose metabolism. GR, a Type I steroid hormone receptor, is a central regulator of energy homeostasis, modulating gluconeogenesis, insulin sensitivity, adipose tissue distribution, and lipid mobilization in response to stress and circadian cues. Dysregulated GR signaling contributes to insulin resistance, visceral obesity, dyslipidemia, and hepatic steatosis, linking chronic glucocorticoid exposure to metabolic syndrome and cardiometabolic disease. RXRs serve as obligate heterodimeric partners for many Type II NRs, including PPARs, LXRs, FXR, TRs, and VDR, acting as integrative hubs that coordinate lipid, glucose, cholesterol, and bile acid metabolism across tissues. RXR ligand availability and permissive versus non-permissive heterodimer behavior critically influence downstream transcriptional outcomes, highlighting RXRs as active modulators of metabolic network integration rather than passive binding partners. Consequently, alterations in RXR signaling can amplify or constrain the metabolic actions of multiple partner receptors simultaneously, underscoring their relevance in metabolic disease pathophysiology and therapeutic targeting [20,21,22].

Metabolic NRs, including peroxisome proliferator-activated receptors (PPARs), LXRs, farnesoid X receptor (FXR), pregnane X receptor (PXR), thyroid receptors (TRs), and estrogen-related receptors (ERRs), belong to Type II NRs and serve as key regulators of lipid uptake, fatty acid oxidation (FAO), cholesterol homeostasis, bile acid synthesis, and clearance pathways. These receptors form heterodimers with retinoid X receptors (RXRs) and directly bind to hormone response elements (HREs) on DNA to modulate gene transcription, even in the absence of ligands [22]. This mechanism enables Type II receptors to maintain precise transcriptional control, fine-tuning multiple physiological processes. Type II receptors are crucial not only for maintaining metabolic homeostasis but also for supporting many other important physiological processes [23]. The potential of these to recruit corepressors and coactivators further refines transcriptional regulation, positioning them centrally in both normal physiology and disease pathogenesis. Dysregulation has been linked to metabolic disorders, neurodegenerative diseases, cancer, and inflammatory conditions, underscoring their therapeutic relevance.

Within the metabolic NR family, PPARα promotes hepatic FAO, PPARγ orchestrates adipogenesis and insulin sensitivity, FXR governs bile acid metabolism, and LXR regulates cholesterol turnover. Disruption of these pathways contributes to insulin resistance, dyslipidemia, and hepatic steatosis. Clinically, several approved drugs act via metabolic NRs: fibrates (PPARα agonists) for hyperlipidemia, thiazolidinediones (PPARγ agonists) for T2D, while FXR agonists remain in development for MASH. Achieving receptor selectivity while minimizing adverse effects remains challenging, prompting the development of alternative ligand strategies such as partial agonists and selective PPAR modulators (SPPARMs). These recruit coactivators less strongly than full agonists, preserving metabolic efficacy while reducing side effects, including weight gain, edema, and cardiovascular complications [24,25,26,27]. Beyond classical metabolic receptors, emerging NRs (TRβ, ERRα, LXRα/β) are increasingly recognized for roles in lipid and glucose metabolism, mitochondrial biogenesis, and energy expenditure. These receptors represent promising therapeutic targets for MAFLD/MASH, obesity, and T2D. Understanding their structure-function relationships, ligand specificity, and tissue-selective actions may enable the development of safer, more effective metabolic therapies [28,29].

The purpose of this review is to present a critical overview of recent advances in selective pharmacological modulation of TRβ, ERRα, and LXRα/β, focusing on their therapeutic potential for treating metabolic disorders (MAFLD, obesity, dyslipidemia). We examine small-molecule modulator development, evaluate preclinical and clinical efficacy, and discuss translational prospects for precision medicine in reprogramming dysregulated metabolic pathways.

## 2. Structural Biology and Activation Mechanisms of NRs

### 2.1. A Common Blueprint

Although NRs regulate an extraordinarily broad spectrum of physiological functions, nearly all superfamily members share a conserved modular domain organization. This common structural framework underpins their remarkable versatility. The modular architecture, where distinct regions specialize in ligand sensing, DNA recognition, or engagement with transcriptional coregulators, enables NRs to detect chemical signals and convert them into precisely calibrated transcriptional outputs. Over evolutionary time, this blueprint has been refined to balance the structural rigidity needed for recognizing specific DNA response elements with localized flexibility in domains mediating ligand binding and cofactor recruitment [20,30,31]. Consequently, even subtle sequence changes or conformational adjustments within a single domain can markedly influence receptor behavior across the family. Thus, this “shared blueprint” functions not only as a structural definition but also as a conceptual lens for understanding how NRs integrate molecular cues into coordinated gene regulatory programs.

**Figure 1:**
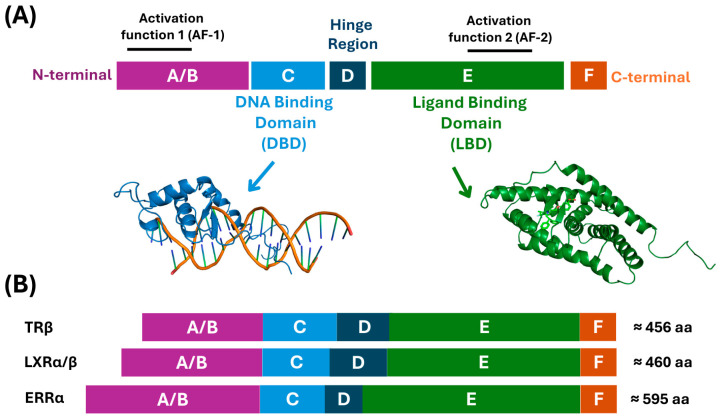
Modular domain organization of NRs. (**A**) NRs exhibit a conserved blueprint characterized by an unstructured N-terminal region (A/B) containing the AF-1 surface, a central DBD, a flexible hinge region (D), and an LBD that accommodates ligands and interacts with coregulatory proteins through the AF-2 surface. This canonical organization does not apply to all NRs, as some atypical members (e.g., Rev-Erbα/β) lack helix 12 and a functional AF-2 domain. (**B**) Domain dimensions and amino acid lengths of TRβ, LXR, and ERRα.

Structurally, NRs exhibit several hallmark modules [20,32] (Figure 1). The DNA-binding domain (DBD), the most highly conserved region within the NR superfamily, identifies hormone response elements (HREs) in target genes. The C-terminal ligand-binding domain (LBD) binds small lipophilic ligands and governs receptor activation, dimerization, and coregulator interactions [33,34]. The LBD also contains the activation function-2 (AF-2) surface, formed by helices 3, 4, and 12 [20,35], although this structural arrangement is not universal across the NR superfamily. Helix 12, also termed the activation function helix (AF-H), is conformationally dynamic, shifting in response to ligand binding to reposition AF-2, enabling selective interactions with co-regulatory proteins. Notably, atypical NRs such as Rev-Erbα/β lack a canonical helix 12 and functional AF-2 surface and therefore act predominantly as transcriptional repressors. In contrast, the N-terminal AF-1 domain can modulate transcription independently of ligand binding [36].

NRs predominantly pair with RXR to form heterodimers. In the absence of ligand, corepressors maintain target genes in repressed configurations. Ligand binding triggers conformational rearrangements promoting coactivator recruitment, shifting receptors into transcriptionally active states. This ligand-dependent switch allows NRs to fine-tune metabolic gene networks in response to hormonal and nutritional signals.

### 2.2. Molecular Basis of Ligand Binding and Transactivation

The molecular basis of ligand-dependent transactivation in NRs relies on a conserved conformational switch within the LBD. Agonist engagement activates the receptor, which binds HREs to control gene transcription. Ligand binding stabilizes the hydrophobic LBD pocket and triggers coordinated rearrangement of its α-helical framework, culminating in helix 12 repositioning. Helix 12, also termed AF-2, is essential for recruiting coactivator proteins [37] in NRs that possess a canonical AF-2 domain.

In the agonist-bound conformation, helix 12 folds over the ligand-binding pocket and, together with helices 3 and 4/5, forms a charged interaction surface accommodating coactivators through characteristic LXXLL motifs, also known as NR boxes. These are short α-helical peptide motifs consisting of a conserved Leu-X-X-Leu-Leu sequence [38]. These motifs create hydrophobic interfaces allowing coactivators from multiple families, including members of the p160/SRC family (SRC-1, SRC-2, SRC-3), metabolic coactivators such as PGC-1α, and additional coregulators such as RIP140, TRAP/Mediator complex subunits, and ASC1/2 to bind selectively to activated receptors.

The LBD also mediates higher-order assembly processes (dimerization, tetramerization) critical for high-affinity DNA regulatory element binding. A flexible hinge region connects the LBD to the DBD; this segment may contain nuclear localization signals (NLS) in several, but not all, NRs, and influences intracellular trafficking and subcellular distribution of hormone-receptor complexes.

Once recruited, coactivators, many possessing histone acetyltransferase (HAT) activity or acting as scaffolds for chromatin-remodeling complexes, promote chromatin relaxation, thereby allowing efficient assembly of the transcriptional machinery at promoter regions [39,40]. In many NRs, ligand binding functions as a structural trigger converting receptors from inactive to active states; however, some receptors, such as estrogen-related receptors (ERRs), display constitutive transcriptional activity even in the absence of endogenous ligands.

In many receptors, ligand absence results in cytoplasmic sequestration by molecular chaperones (heat shock protein 90 [HSP90], HSP70), maintaining receptors in inactive configurations, a well-established mechanism for several Type I steroid receptors (e.g., glucocorticoid and mineralocorticoid receptors), where the chaperone complex stabilizes the unliganded receptor and masks nuclear localization signals prior to ligand binding. However, this cytoplasmic retention is not a universal feature of all NRs: many Type II receptors (such as TR, RAR, and RXR) are predominantly localized to the nucleus regardless of ligand status and are regulated mainly through dynamic corepressor/coactivator exchange at DNA response elements.

**Figure 2:**
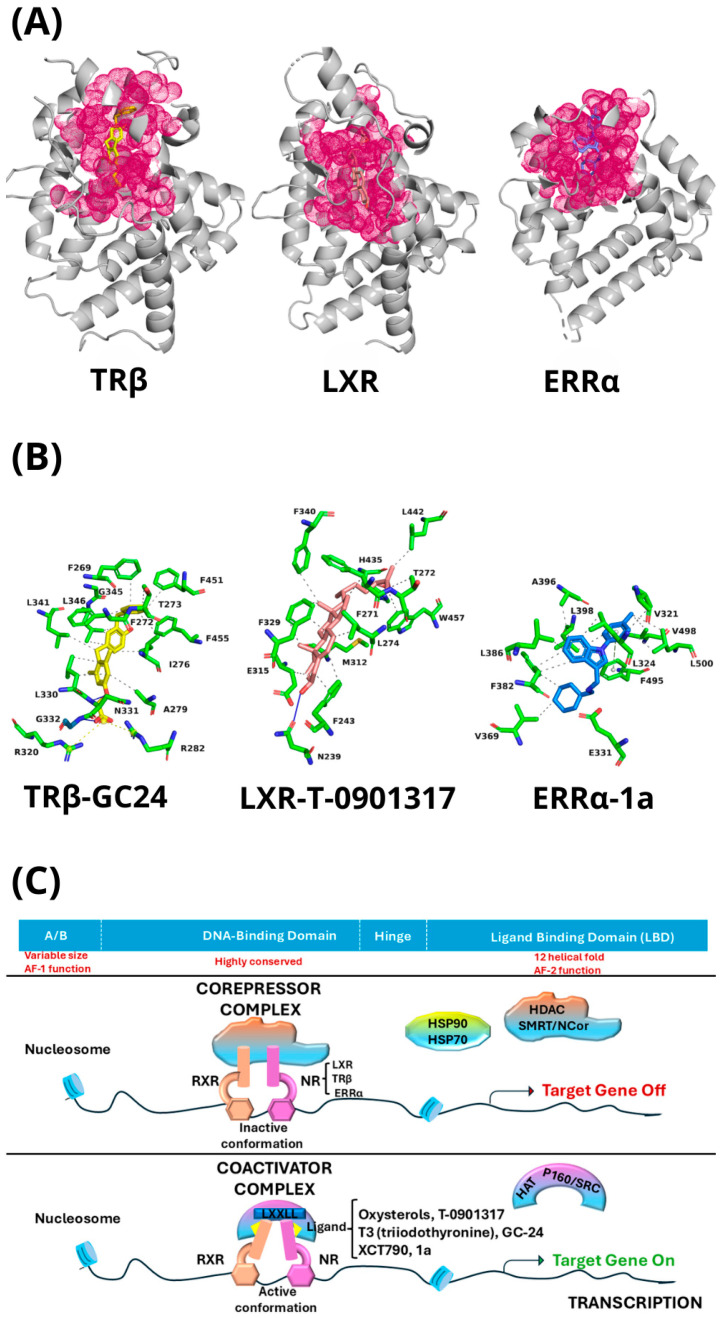
Structural basis of target recognition and activation. (**A**) Comparison of ligand-binding cavities in TRβ (PDB: 1Q4X), LXR (PDB: 1P8D), and ERRα (PDB: 2PJL), represented as Connolly surfaces to highlight differences in pocket size, depth, and topology. (**B**) Close-up views of the TRβ LBD–GC-24 complex (PDB: 1Q4X), LXR LBD–T-0901317 complex (PDB: 1P8D), and ERRα-compound **1a** complex LBD (PDB: 2PJL). The ligand–receptor complexes are oriented consistently with panel A to facilitate direct comparison between binding-pocket architecture and residue–ligand interactions. Key pocket-lining residues interacting with the ligands are labeled in this panel. Hydrogen bonds are shown as blue lines, salt bridges as yellow dashed lines, and hydrophobic contacts as gray dashed lines. Aromatic interactions (π-π stacking) are indicated by dots at aromatic ring centers. (**C**) Schematic representation of the NRs transactivation mechanism.

A representative diagram of the transactivation mechanism appears in Figure 2.

### 2.3. Comparative Structural Analysis of TRβ, ERRα, and LXR

The LBDs of TRβ, ERRα, and LXR display distinct structural features determining ligand specificity and functional modulation modes. TRβ contains a relatively small, elongated binding pocket optimized for thyroid hormone T3; key residues (S277, R320, H435) establish hydrogen bonds enabling selective recognition, while surrounding hydrophobic residues stabilize the ligand’s aromatic core [41]. The crystal structure of human TRβ LBD at 2.8 Å resolution complexed with GC-24, a triiodothyronine (T3) analog, clarifies the basis of its agonism and specificity. Borngraeber et al. [41] demonstrated that GC-24’s benzyl group is held through 3–4 Å shifts in two helices essential for hormone binding and correct helix 12 C-terminal positioning. Although these rearrangements occur, the complex binds coactivators as closely as the TRβ–T3 complex, remaining fully active. The findings indicate that enhanced ligand specificity stems from a newly formed hydrophobic cluster involving both ligand and protein residues. Interestingly, other thyromimetics where iodine is replaced with bulky substituents improve TR-β1 selectivity. This selectivity is particularly relevant given the distinct biological roles of TRβ isoforms: TRβ1 is widely expressed in metabolically active tissues such as liver, kidney, and skeletal muscle, whereas TRβ2 expression is largely restricted to the hypothalamus, pituitary, cochlea, and retina, where it plays a key role in hypothalamic–pituitary–thyroid axis regulation and sensory development. The X-ray crystal structure of compound **15c** bound to TR-β1 LBD [42] shows M442 displacement by the bulky R3′ phenyl-ethyl-amide side [42] chain. This conformational shift expands the binding pocket to accommodate the substituent, while the ligand–receptor complex preserves full agonist activity. Importantly, reduced activity toward TRβ2 is desirable to minimize central and endocrine side effects, reinforcing the therapeutic value of TRβ1-selective agonists for metabolic disorders.

In contrast, ERRα possesses a larger, more flexible ligand-binding pocket that accommodates both agonists and inverse agonists. Residues (L268, I310, F435) contribute extensive hydrophobic and π–π interactions, enabling conformational adjustments, particularly within helix 12 (H12), thereby modulating coactivator recruitment. The receptor naturally adopts an active conformation even in the absence of ligand, with its LBD positioning H12 in an “agonist-like” state, favoring coactivator interactions (PGC-1α, SRC-1). Inverse agonists bind deep within the LBD cavity, inducing pocket-lining residue reorientation and H12 misalignment, disrupting canonical coactivator-binding groove formation and strongly reducing constitutive transcriptional activity.

Most inverse agonists exhibit shared binding modes, targeting residues in H3, H5, the H6/H7 loop, and H11 [43]. According to Karnati et al. [43], common hot-spot residues strongly interacting with inverse agonists include L324, F328, F382, L398, F495, and L500. For effective inverse agonism, strong binding to the aromatic cluster formed by F328 (H3), F495 (H11), F382 (H5/H6 loop), alongside L500, proves essential for stabilizing inactive receptor conformations. In the crystal complex with the inverse agonist cyclohexylmethyl-(1-p-tolyl-1H-indol-3-ylmethyl)-amine (compound **1a**) [44], the receptor undergoes pronounced conformational changes within its ligand-binding pocket (LBP) to create sufficient space for ligand accommodation. These rearrangements cause F328 on helix H3 to adopt a new side-chain conformation, forcing F510 on H12 to shift and thereby displacing the activation helix from its agonist position. This process reveals a distinct mechanism of helix 12 inactivation, differing from those previously described for ERR, estrogen receptor (ER), and related NRs. Notably, the displaced H12 relocates into the coactivator-binding groove, where it adopts a conformation that closely mimics a bound coactivator peptide.

Liver X receptors (LXRs) feature broad, deep, predominantly hydrophobic ligand-binding pockets optimized for oxysterols. Structural analyses of protein-ligand complexes obtained by X-ray crystallography [45] indicate that residues H435 and W457 function as an activation switch. H435 displays highly conserved main-chain (φ, ψ) angles, whereas W457 undergoes more pronounced movements. The conserved structural relationship between H435 in helix 11 (H11) and W457 in helix 12 (H12) suggests ligand design promoting LXR activation should focus on induced-fit adjustments primarily around W457, while H435 remains relatively rigid. Additional flexibility arises from the main-chain mobility of F329 and L330, contributing to the LBD’s dynamic nature. R319 shows moderate (φ, ψ) angle changes, further supporting flexible binding cavity notions.

The X-ray crystal structure of the LXRβ LBD complexed with the synthetic agonist T-0901317 [46] shows that this ligand fully occupies the binding pocket, forming extensive lipophilic contacts and one key hydrogen bond to H435, helping to maintain the receptor in an active conformation [46]. AF-2 region recruitment occurs not through direct polar interactions between ligand and AF-2 side chains but rather through hydrophobic contacts and, notably, through indirect mechanisms that pre-orient side chains around the ligand binding pocket, shaping the interface with helix AF-2 [46].

Consistently, LXRβ structures bound to synthetic ligands T0901317 and GW3965 [47] reveal highly flexible binding pockets adapting to structurally distinct ligands. The cavity is predominantly hydrophobic yet contains polar or charged residues at its two ends. T0901317 exploits this by forming interactions with H435 near H12 [46] while GW3965 positions its charged group toward the opposite end. Despite different orientations, both ligands induce the characteristic “agonist conformation” of H12, leading to a transcriptionally active receptor. Comparative analysis highlights that while TRβ relies on precise polar interactions within a rigid pocket, ERRα leverages plasticity and hydrophobic packing, and LXR balances cavity volume and hydrophobicity to accommodate diverse sterol ligands. These structural distinctions rationalize differences in ligand selectivity and mechanistic bases for receptor activation or inhibition, providing frameworks for targeted ligand design. Table 2 summarizes distinctive features of TRβ, ERRα, and LXR ligand-binding pockets together with corresponding X-ray crystal structures, while Figure 2A,B illustrate binding pockets and LBD–ligand interactions discussed above.

## 3. Thyroid Hormone Receptor β (TRβ) Agonists: Redirecting Hepatic Metabolism

### 3.1. TRβ and Hepatic Lipid Metabolism

Thyroid hormones (THs) are synthesized in the thyroid gland, which consists of follicles where tyrosine residues within the glycoprotein thyroglobulin are iodinated [51,52]. TH actions are mediated by two receptor genes, *TRalpha* and *TRbeta*, with distinct expression profiles during development and in adult tissues [53]. TRα1 predominates in the brain, heart, and skeletal muscle, whereas TRβ1 is expressed in multiple tissues, including the liver, kidney, brain, and spleen, but represents the major TRβ isoform functionally relevant for hepatic lipid and cholesterol metabolism. This functional predominance in hepatocytes, rather than liver-restricted expression, underlies the therapeutic focus on TRβ1 in metabolic disease. Unless otherwise specified, in the context of this review, the term TRβ refers to the TRβ1 isoform.

In hepatocytes, TRβ enhances low-density lipoprotein receptor (*LDLR*) expression, thereby promoting cholesterol clearance, stimulates bile acid synthesis via *CYP7A1*, upregulates genes involved in fatty acid β-oxidation (*CPT1α*, *ACOX1*), supports mitochondrial biogenesis, and represses lipogenic transcription factors such as sterol regulatory element-binding protein-1c (SREBP-1c) [54,55]. TRβ also improves hepatic insulin sensitivity and glucose turnover. These coordinated metabolic actions define the physiological framework underlying the therapeutic targeting of TRβ discussed in the following sections. Because TRβ is also expressed in extrahepatic tissues, including the central nervous system, kidneys, and endocrine organs, substantial efforts have focused on the development of liver-selective TRβ agonists and prodrug strategies to maximize hepatic metabolic benefits while minimizing systemic thyromimetic effects. At the molecular level, TRβ functions as a ligand-activated NR. Upon binding T_3_ or selective TRβ agonists, it heterodimerizes with RXR and interacts with thyroid hormone response elements (TREs) in target gene promoters. In the absence of ligand, TRβ associates with corepressors (NCoR, SMRT) to maintain transcriptional repression. Ligand binding induces conformational changes, displacing corepressors and recruiting coactivators (SRC-1, PGC-1α, CBP/p300), which remodel chromatin and initiate transcription [54,56]. Through this mechanism, TRβ agonists regulate hepatocyte metabolic pathways, promoting cholesterol clearance, stimulating fatty acid oxidation (FAO), and suppressing lipogenesis. THs further coordinate hepatic lipid metabolism by mobilizing free fatty acids (FFAs) from adipose tissue, enhancing adipogenesis via fatty acid synthase (*FASN*), promoting triacylglycerol (TAG) esterification and very low-density lipoprotein (VLDL) packaging, and stimulating lipolysis and lipophagy. They increase mitochondrial biogenesis and β-oxidation, shift phospholipid metabolism toward phosphatidylcholine synthesis via *Pcyt1*, *Pcyt2*, and *PEMT*, and inhibit phospholipid hydrolysis, thereby reducing TAG accumulation. Elevated TH levels favor net hepatic lipid clearance independently of thyroid status [29,55,57,58,59,60].

### 3.2. TRβ as a Therapeutic Target in MASH and Dyslipidemia

Given its central role in hepatic metabolism, TRβ represents a promising therapeutic target for MAFLD, MASH, and dyslipidemia. Impaired TRβ signaling contributes to triglyceride and cholesterol accumulation in hepatocytes, oxidative stress, and inflammation, hallmarks of these conditions [61,62,63,64]. TRβ activation enhances cholesterol clearance, stimulates FAO, reduces lipogenesis, and exerts anti-inflammatory and anti-fibrotic effects by modulating oxidative stress and inflammatory cytokine pathways [63]. Preclinical studies confirm liver-specific TRβ functions: in TRβ-deficient mice, *CYP7A1* is unresponsive to T_3_, and cholesterol metabolism remains impaired, whereas TRα1-deficient mice retain normal hepatic cholesterol handling [65]. Therapeutic TRβ1 agonists improve systemic lipid profiles by upregulating *LDLR* expression and enhancing cholesterol clearance, while minimizing off-target effects on heart and bone, which are mainly TRα-regulated [66]. Consistent with its physiological role described above, key steps under TRβ1 control include *LDLR*-mediated cholesterol clearance, HMG-CoA reductase-dependent cholesterol biosynthesis, and *CYP7A1*-mediated bile acid synthesis [61,62]. Moreover, hypothyroidism has been linked to increased hepatocellular carcinoma (HCC) risk, while T_3_ administration reduces tumor progression and metastasis [67,68,69]. Taken together, these findings justify the development of selective TRβ1 agonists to harness beneficial hepatic TH effects while minimizing systemic and cardiac side effects, providing potential therapeutic strategies for MAFLD, MASH, and dyslipidemia [29,55,57,58,59,60,61,62,63,64,65,66,67,68,69].

### 3.3. Clinical and Preclinical Landscape of TRβ Agonists: Thyromimetics and Emerging Hybrid Compounds

The therapeutic potential of TRβ agonists for treating MAFLD, MASH, and dyslipidemia derives from liver-enriched TRβ expression and its pivotal role in regulating hepatic lipid and energy metabolism.

Building on the mechanistic and pathological rationale outlined in Section 3.1 and Section 3.2, this section summarizes the clinical and preclinical development of TRβ-targeted therapies.

Most TRβ agonists are thyromimetics, designed to mimic natural TH T3 by binding to the TRβ LBD, thereby selectively modulating transcription of genes involved in fatty acid β-oxidation, cholesterol catabolism via *LDLR* and *CYP7A1*, lipoprotein clearance, mitochondrial biogenesis, and lipophagy, while minimizing off-target effects mediated by TRα in heart, skeletal muscle, and bone.

**Figure 3:**
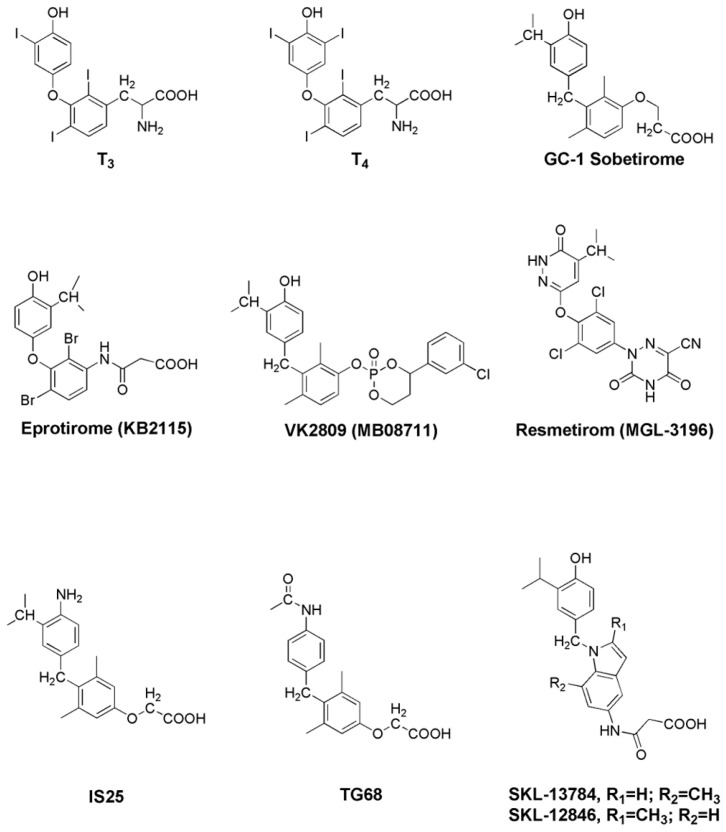
Chemical structures of TRβ-selective thyromimetics compared with physiological ligands T3 and T4.

**Sobetirome **(**GC-1**), 3,5-dimethyl-4-(4′-hydroxy-3′-isopropylbenzyl)phenoxy acetic acid (Figure 3), differs from T3 by containing three hydrocarbon residues replacing iodine atoms, a methylene linkage between phenyl rings, and substitution of 1-aminopropionic acid with oxyacetic acid. This synthetic TH analog belongs to the first generation of TRβ-specific agonists, exhibiting high liver accumulation and a 10-fold lower TRα affinity than T3 [70]. In preclinical MASH and obesity models, **GC-1** reduced hepatic steatosis, liver lipid peroxidation, serum triglycerides and cholesterol, and improved liver injury markers (AST, ALT), while sparing heart and bones [71,72,73]. In phase 1 studies, GC-1 reduced LDL cholesterol (*LDL-C*) by 41% over 2 weeks in healthy volunteers; however, further development was discontinued due to lack of funding.

**GC-24**, a second-generation analog with enhanced TRβ selectivity (40-fold TRβ:TRα binding), improved plasma triglycerides, body fat, glucose tolerance, and insulin sensitivity in high-fat diet (HFD) rats but failed to restore hypercholesterolemia and had lower hepatic targeting than GC-1 or T3 [74].

**Eprotirome **(**KB2115**)**, **3-[[3,5-dibromo-4-[4-hydroxy-3-(1-methylethyl)-phenoxy]-phenyl]-amino]-3-oxopropanoic acid (Figure 3), a brominated TRβ agonist, demonstrated high liver selectivity, reduced hepatic steatosis and serum LDL in ob/ob and HFD rats, and decreased cholesterol in human subjects with dyslipidemia [73,75]. Early clinical trials (NCT00593047, NCT01410383) [76,77] showed LDL reductions of approximately 40% without adverse cardiac effects; however, a phase 3 trial was terminated due to cartilage toxicity in canine studies [78].

**VK2809** (**MB07811**) (2*R*, 4*S*)-4-(3-chlorophenyl)-2-[(3,5-dimethyl-4-(4′-hydroxy-3′-isopropylbenzyl) phenoxy) methyl]-2-oxido-[1, 3, 2]-dioxaphosphonane) (Figure 3), is an orally active liver-targeted prodrug converted to MB07344 in hepatocytes, producing strong liver-first-pass effects with minimal systemic exposure [79]. In preclinical models, **VK2809** reduced hepatic steatosis, triglycerides, and serum FFAs, increased β-oxidation and mitochondrial respiration, and spared cardiac and skeletal tissues [80]. Phase 1 and 2 studies showed dose-dependent reductions in LDL-C, triglycerides, and liver fat content (median reductions 53–60% by magnetic resonance imaging-proton density fat fraction [MRI-PDFF]), with no significant alanine aminotransferase (ALT) elevation after 12 weeks [81]. A phase 2b study (NCT04173065) in 337 biopsy-confirmed MASH patients confirmed sustained liver fat reduction after 52 weeks. A recent 52-week follow-up study demonstrated that **VK2809** (5 mg and 10 mg daily) achieved significant histological improvements in MASH resolution and fibrosis regression, with favorable safety profiles [https://pmc.ncbi.nlm.nih.gov/articles/PMC11784560/, accesses on 9 December 2025]. A four-week post-treatment follow-up of the 12-week phase 2 randomized, placebo-controlled study showed that **VK2809** continued to reduce liver fat in patients with MAFLD.

**Resmetirom **(**MGL-3196**), a liver-targeted TRβ agonist (Figure 3) featuring a cyanoazauracil substituent, shows approximately 28-fold greater TRβ selectivity compared with T3 [75]. This structural modification markedly enhances potency and specificity, enabling the compound to reduce hepatic triglycerides, steatosis, lipid peroxidation, and inflammatory and fibrotic markers in animal MASH models [82]. In phase 2 trials, **resmetirom** reduced liver fat by 32.9–37.3% versus 8.5–10.4% in placebo at 12–36 weeks, decreased atherogenic lipids (LDL-C, apolipoprotein B [ApoB], triglycerides), and improved MASH biopsy scores in 56% of patients [83,84]. The pivotal phase 3

MAESTRO–MASH trial confirmed histological improvement and significant liver fat reduction with favorable safety at 80–100 mg/day for treating MASH with liver fibrosis [85]. In March 2024, **resmetirom **(**Rezdiffra™**) received FDA approval for the treatment of MASH with moderate-to-advanced liver fibrosis (F2-F3), representing the first approved therapy specifically for MASH [86]. This landmark approval validates TRβ as a therapeutic target for metabolic liver disease and establishes a new standard of care for MASH patients.

**IS25** and its prodrug **TG68** are halogen-free TRβ agonists based on GC-1 scaffolds (Figure 3) [87]. TG68 reduced lipid accumulation in HepG2 cells via AMP-activated protein kinase (AMPK) activation and acetyl-CoA carboxylase (ACC) inhibition. In F344 rats and HFD-fed C57BL/6 mice, TG68 decreased hepatic steatosis, triglycerides, serum transaminases, and liver weight without extrahepatic toxicity [88].

Indole-based agonists **SKL-12846** and **SKL-13784** (Figure 3) demonstrated high liver specificity and cholesterol-lowering activity in preclinical models with minimal cardiac effects, highlighting indole scaffolds as promising TRβ-selective chemotypes [89,90].

Beyond classical thyromimetics, a novel **Glucagon/T3** **hybrid** combines the anti-lipid effects of glucagon with energy-expending actions of T3. In obese mice, this hybrid reduced adipose mass, reversed MASH, ameliorated dyslipidemia, decreased atherosclerotic plaque formation, and improved glucose metabolism while avoiding thyrotoxicosis and diabetogenic glucagon effects, representing a promising alternative to isoform-selective TRβ agonists [63,91]. Collectively, these preclinical and clinical studies demonstrate that liver-targeted TRβ activation, whether via thyromimetics or engineered co-agonists, effectively modulates hepatic lipid metabolism, reduces liver fat, improves systemic lipid profiles, and mitigates inflammation and fibrosis, providing potent pharmacological strategies for MAFLD, MASH, and dyslipidemia while minimizing systemic thyrotoxic effects. In Table 3, the pharmacological and clinical profiles of TRβ agonists are reported.

## 4. Estrogen-Related Receptor α (ERRα) Modulators: Reprogramming Mitochondrial Function

### 4.1. Physiological Role and Molecular Networks

Estrogen-related receptor α (ERRα) is an orphan NR structurally related to classical estrogen receptors but does not bind endogenous estrogens. ERRα serves as a central regulator of energy metabolism, particularly in high-energy-demand tissues (skeletal muscle, heart, liver, brown adipose tissue). Functionally, ERRα plays key roles in mitochondrial biogenesis and oxidative phosphorylation (*OXPHOS*) [28]. Through cooperation with coactivators such as PGC-1α, ERRα promotes transcription of genes encoding mitochondrial transcription factor A (TFAM) and electron transport chain (ETC) components, thereby increasing mitochondrial number and respiratory capacity. Beyond mitochondrial regulation, ERRα contributes to energy homeostasis, modulating FAO, glucose metabolism, and ATP production. In skeletal muscle, ERRα activation supports an oxidative phenotype and improves endurance capacity [92]. It also mediates metabolic flexibility, enabling cells to switch between carbohydrate and fatty acid utilization in response to varying energy demands, often coordinating with AMPK signaling pathways. Dysregulated ERRα activity has been implicated in metabolic disorders, CVD, and age-related mitochondrial decline, highlighting its therapeutic potential [92].

At the molecular level, ERRα functions within complex transcriptional networks, forming a feed-forward loop with PGC-1α/β, which recruits ERRα to promoters of genes involved in the ETC, tricarboxylic acid (TCA) cycle, and FAO. ERRα also interacts with other NRs (PPARs, nuclear respiratory factors [NRFs], LXR), allowing fine-tuning of metabolic gene expression. Absent activating signals, ERRα associates with corepressors (NCoR1, SMRT), suppressing mitochondrial and metabolic gene transcription, a mechanism exploited by inverse agonists to stabilize repressive conformations. Moreover, ERRα activity is modulated by nutrient availability, hormonal cues, cellular stress, and post-translational modifications (phosphorylation, acetylation), integrating multiple signals to maintain cellular energy balance.

ERRα’s hepatic role has been increasingly recognized. Rui et al. and Moore et al. describe ERRα as a major transcriptional regulator of hepatocellular metabolism, controlling gene networks governing liver energy homeostasis and mitochondrial function [93,94]. Despite structural similarity to estrogen receptors, ERRα is not activated by estrogens and remains an orphan receptor, making it unique among NRs [95,96]. Studies combining transcriptomics, genome-wide chromatin immunoprecipitation (ChIP), and ERRα-null mouse models have revealed its essential role in liver metabolism, including insulin sensitivity regulation, lipid handling, and prevention of metabolic diseases (MAFLD, T2D) [97]. This highlights ERRα as a sensor of intrinsic and environmental cues, with activity modulation representing a promising strategy to improve liver function and systemic metabolic health.

### 4.2. Pharmacological Targeting of ERRα

ERRα is a critical transcriptional regulator in the liver, orchestrating mitochondrial function, oxidative metabolism, and lipid homeostasis by integrating nutrient, hormonal, and stress-derived signals [97]. This central role links hepatic ERRα not only to normal systemic energy balance, coordinating liver metabolism with that of skeletal muscle, adipose tissue, gut, and brain, but also to multiple pathological states [98].

In insulin resistance and T2D, dysregulated ERRα-mediated transcription of oxidative phosphorylation and mitochondrial genes has been associated with altered mitochondrial function in skeletal muscle, where reduced PGC-1α/ERRα activity correlates with decreased OXPHOS gene expression and impaired oxidative capacity that contributes to metabolic dysfunction. In this context, pharmacological activation of ERRα can restore mitochondrial gene expression, enhance oxidative metabolism, and improve insulin sensitivity.

In MAFLD, ERRα effects are context-dependent: under HFD conditions, its upregulation appears adaptive by promoting mitochondrial compensation, whereas its deficiency impairs recovery from fasting-induced steatosis and may worsen hepatic lipid accumulation. These findings indicate that ERRα supports metabolic flexibility under lipid overload but may also drive maladaptive programs when chronically activated.

Pharmacologically, both ERRα activation and inhibition have been explored as therapeutic strategies, with beneficial effects depending on the disease context. Activation of hepatic ERRα improves insulin sensitivity and reduces fat in MAFLD models, whereas inhibition can reduce lipid biosynthesis and attenuate steatohepatitis by downregulating genes such as *GPAT4* [99]. Thus, ERRα agonists primarily enhance mitochondrial oxidative capacity, while inverse agonists suppress specific anabolic and lipogenic transcriptional circuits.

ERRα also regulates VLDL assembly and secretion in a sex-dependent manner via target genes including *ApoB*, *MTTP*, and *Pla2g12b* [100]. In HCC, ERRα modulates tumor metabolism, ROS production, and inflammation through NF-κB, suggesting therapeutic potential in liver cancer. Collectively, these observations explain why both ERRα agonists and inverse agonists can exert beneficial metabolic effects, depending on tissue context, disease stage, and dominant transcriptional pathways engaged, highlighting ERRα as a highly versatile and context-sensitive pharmacological target.

### 4.3. Promising ERRα Modulators in Development

**Figure 4:**
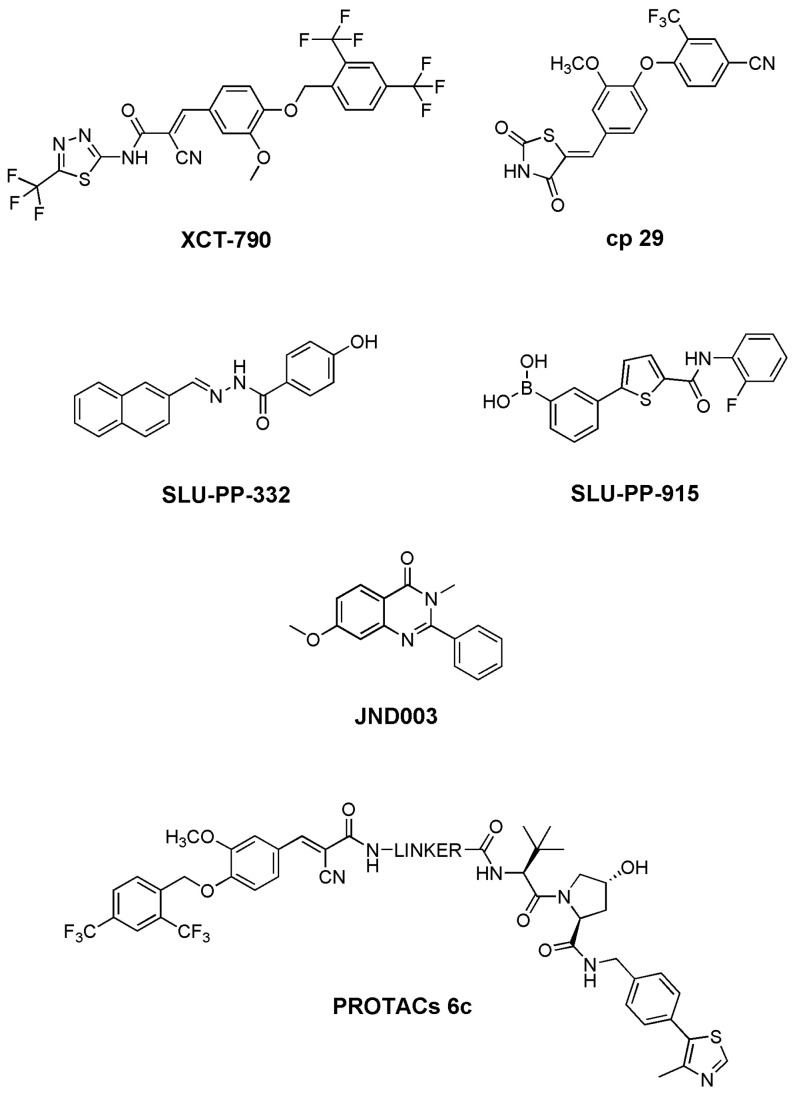
Chemical structures of ERRα modulators in development.

Over the past two decades, a broad array of small-molecule modulators targeting ERRα has been developed, enabling increasingly precise pharmacological control over metabolic functions regulated by this NR. Early inverse agonists such as **XCT-790**, (2*E*)-3-(4-{[2,4-bis(trifluoromethyl)phenyl]methoxy}-3-methoxyphenyl)-2-cyano-N-[5-(trifluoromethyl)-1,3,4-thiadiazol-2-yl]prop-2-enamide (Figure 4) first demonstrated that ERRα-driven transcriptional programs could be chemically downregulated, although their effects were complicated by off-target activities, including mitochondrial uncoupling [101]. Subsequent availability of high-resolution X-ray crystal structures of the ERRα LBD provided detailed structural frameworks that facilitated the rational design of more selective modulators with reduced mitochondrial toxicity [43].

Recent studies have highlighted the broader physiological relevance of ERRα, particularly in angiogenesis associated with proliferative diabetic retinopathy (PDR). One study [102] demonstrated that the PGC-1α/ERRα signaling axis contributes to regulating angiogenic factor expression in PDR. Using human vitreous samples, fibrovascular epiretinal membranes, diabetic rat retinas, and cultured human retinal Müller glia and endothelial cells, the authors showed PGC-1α and ERRα are co-expressed in endothelial cells and leukocytes within PDR membranes, with levels positively correlating with angiogenic activity alongside vascular endothelial growth factor (VEGF) and angiopoietin-2, markedly elevated in both PDR vitreous and diabetic rat retinas. In Müller cells, pharmacological inhibition of either PGC-1α or ERRα attenuated diabetes-mimetic induction of VEGF, angiopoietin-2, and monocyte chemoattractant protein-1/C-C motif chemokine ligand 2 (MCP-1/CCL2), whereas PGC-1α activation increased VEGF expression and reduced ROS levels, indicating that the PGC-1α/ERRα pathway plays supportive roles in promoting angiogenic factor upregulation. Beyond angiogenesis, ERRα modulators have important implications for metabolic regulation, including glucose uptake, dyslipidemias, and MASH (Table 4).

In this context, a diaryl ether-based thiazolidinedione, compound **29** [103] (Figure 4), has been described as a selective, potent ERRα antagonist with favorable pharmacokinetic properties, representing a promising candidate for metabolic disorders. Molecular modeling and docking studies suggested compound **29** binds tightly to the ERRα LBD, forming stable hydrogen bonds with residues Glu235 and Tyr326 and hydrophobic interactions with Leu228, Phe232, and Ile349, critical for receptor activation and ligand selectivity. These structural insights likely explain both its high affinity and selectivity toward ERRα over related NRs.

Metabolic and antidiabetic effects were evaluated in four preclinical studies using mouse and rat obesity and diabetes models. In AKR/J mice, thirty males fed HFD (45% kcal from fat) received compound **29** (3 or 30 mg/kg, twice daily) for 5 days, with measurements of food intake, body composition, plasma insulin, liver function, and tissue collection. In C57BL/6 mice, forty males on 60% HFD were treated with compound **29** (10 or 30 mg/kg, once daily) for 18 days, monitoring body weight, composition, glucose, lipid profiles, insulin, and oral glucose tolerance test (OGTT); ten low-fat diet mice served as controls [103]. In Zucker diabetic fatty (ZDF) fa/fa rats, two studies were conducted: a 12-day study where fifty males received compound **29** (10 mg/kg) or controls, assessing body weight, food intake, and insulin tolerance testing (ITT), and a 25-day dose–response study in sixty-five males treated with compound **29** at 0.08–10 mg/kg, monitoring body weight, food intake, glucose, insulin, lipid levels, and OGTT performance. Across all studies, tissues were collected for further analysis, with primary endpoints including body composition, glucose homeostasis, insulin sensitivity, lipid metabolism, and dose-dependent metabolic effects [103]. Collectively, these findings, supported by detailed molecular modeling data, suggest selective ERRα antagonism via compound **29** may represent a novel therapeutic strategy for metabolic disorders (obesity, T2D, dyslipidemias) (Table 4). In contrast, whereas early inverse agonists suppressed ERRα, recent efforts have focused on agonists enhancing its transcriptional activity and supporting metabolic homeostasis. Pan-ERR agonists such as **SLU-PP-332 **(4-hydroxy-N-[(Z)-naphthalen-2-ylmethylideneamino]benzamide) [104] and **SLU-PP-915 **[3-[5-[(2-fluorophenyl)carbamoyl]thiophen-2-yl]phenyl]boronic acid (Figure 4), enhance mitochondrial gene expression and oxidative metabolism across all ERR isoforms, mimicking many aerobic exercise effects. In preclinical studies using mouse obesity and metabolic syndrome models, **SLU-PP-332** increased energy expenditure and FAO, reduced fat mass accumulation, and improved insulin sensitivity. These effects align with ERR physiological roles as constitutively active NRs highly expressed in energy-demanding tissues, regulating genes involved in mitochondrial biogenesis and lipid oxidation. Pharmacological ERR activation thus represents a promising strategy to treat obesity and metabolic syndrome, reproducing key metabolic exercise benefits without physical activity [104].

**SLU-PP-915** was developed through a structure-based approach guided by the X-ray structure of compound **1a** bound to the ERRα LBD [44]. Molecular docking studies modeled interactions between **SLU-PP-915** and the ERRα ligand-binding pocket, revealing stable hydrogen bonds with conserved residues (Tyr326, Glu331) and hydrophobic contacts within the protein core. Docking indicated the ligand adopts conformations promoting favorable AF-2 helix rearrangement, consistent with transcriptional activation observed in vitro. These structural insights guided structure-based **SLU-PP-915** optimization, improving both affinity and selectivity, and provided molecular explanations for the rapid in vivo induction of ERR target genes.

In vitro studies in C2C12 myoblasts demonstrated that **SLU-PP-915** [105] robustly upregulates key ERR target genes (*PGC1α*, pyruvate dehydrogenase kinase 4 [*PDK4*], lactate dehydrogenase A [*LDHA*]), critical regulators of mitochondrial biogenesis, FAO, and glucose metabolism. These transcriptional effects were confirmed in vivo, as a single intraperitoneal **SLU-PP-915** dose (20 mg/kg) rapidly increased expression of *PGC1α*, *PDK4*, and the ERRα-specific gene DNA damage-inducible transcript 4 (*DDIT4*) in quadriceps muscle within one hour. Functionally, **SLU-PP-915** enhanced endurance in mice, increasing both running distance and time. Together, these in vitro and in vivo findings indicate that **SLU-PP-915** is a potent ERR agonist capable of activating metabolic gene programs and improving muscle function, highlighting its potential as a therapeutic candidate for metabolic disorders (obesity, insulin resistance, related syndromes) [105] (Table 4).

**JND003** (Figure 4), identified in 2022 through AlphaScreen assays, ref. [99] is a highly selective ERRα agonist. The compound, **7-methoxy-3-methyl-2-phenyl-4(3H)-quinazolinone**, exhibits an EC_50_ of 86.0 nM for promoting ERRα-PGC1α peptide interaction, robustly activating hepatic oxidative pathways, and improving overall metabolic function. Studies in liver-specific ERRα knockout mice demonstrated that ERRα loss exacerbates diet-induced fatty liver, insulin resistance, and impaired glucose disposal, highlighting the receptor’s essential role in liver metabolism [99].

In vitro, **JND003** increased ERRα transcriptional activity, upregulating key target genes (medium-chain acyl-CoA dehydrogenase [*MCAD*], PDK4, ATP synthase subunit beta [*ATP5β*]). In vivo, oral **JND003** administration (10–30 mg/kg/day) in HFD-fed mice improved glucose tolerance, insulin sensitivity, and hepatic steatosis while reducing liver triglycerides and serum ALT/aspartate aminotransferase (AST) levels [99]. The compound also enhanced hepatic FAO and mitochondrial function, reflected by elevated mRNA and protein expression of metabolic genes, including carnitine palmitoyltransferase 1A (*CPT1A*). Pharmacokinetic analysis showed rapid absorption, a short half-life (t_1/2_ = 0.73 h), low oral bioavailability (9%), and pronounced tissue accumulation in the liver and abdominal adipose tissue (up to approximately 59- and 235-fold higher than plasma, respectively). Collectively, these findings support **JND003** as a promising therapeutic candidate for MAFLD and T2D, with beneficial effects primarily mediated through hepatic ERRα activation [99] (Table 4).

Complementing agonists and inverse agonists, in 2019, a series of **(*****E*****)-3-cyanoacrylamide** derivatives designed as proteolysis-targeting chimeras was reported to selectively induce proteasomal degradation of ERRα in cells. One representative compound, **6c** (Figure 4), was shown to decrease ERRα protein levels by >80% at ~30 nM in human cell lines, consistent with PROTAC-mediated target knockdown and providing a useful chemical probe for investigating ERRα functions [106]. While compound **6c** is a valuable research tool for studying ERRα biology, further work is required to define its therapeutic potential in metabolic disease contexts. Table 4 summarizes preclinical modulators of ERRα and their experimental applications.

## 5. Liver X Receptors (LXRα/β) Modulators: Balancing Cholesterol Homeostasis and Lipogenesis

### 5.1. Core Physiological Functions and the LXR Dilemma

Liver X receptors (LXRα/*NR1H3* and LXRβ/*NR1H2*) are oxysterol-activated NRs functioning as central sensors of intracellular cholesterol levels. The human LXRα gene is located on chromosome 11p11.2, whereas LXRβ is found on 19q13.3. Their expression patterns vary widely across tissues: LXRα has a restricted distribution, being highly expressed in the liver and present in the intestine, adipose tissue, macrophages, kidney, and lung. In contrast, LXRβ is ubiquitously expressed and detectable in most examined cell types and tissues [107].

The primary physiological role of LXRs is maintaining cholesterol homeostasis by coordinating gene networks promoting cholesterol efflux, reverse cholesterol transport (RCT), and bile acid synthesis. Ligand-dependent LXR activation induces transcription of ATP-binding cassette transporters (*ABCA1*, *ABCG1*), apolipoprotein A-I (*ApoA-I*), and *CYP7A1*-dependent bile acid synthetic pathway components, collectively reducing intracellular cholesterol accumulation while enhancing systemic clearance. Accordingly, LXR activation promotes cholesterol excretion [108,109] and exerts anti-inflammatory effects by antagonizing NF-κB and activator protein 1 (AP-1) signaling. However, LXR activation simultaneously stimulates de novo triglyceride lipogenesis [110] a potentially deleterious effect leading to hepatic steatosis and insulin resistance [111]. LXRβ, due to ubiquitous expression, plays key roles in immune cells (macrophages), supporting cholesterol efflux and dampening inflammatory activation. LXRα, in contrast, is enriched in liver, intestine, and adipose tissue, exerting stronger control over systemic lipid flux.

Despite their therapeutic potential, LXR agonists face a major limitation known as the “LXR dilemma”: While LXR activation enhances RCT and reduces atherosclerotic plaque burden in preclinical models, it simultaneously upregulates *SREBP-1c*, *FASN*, *ACACA*, and other lipogenic genes, causing hepatic steatosis, hypertriglyceridemia, and insulin resistance. These adverse effects counterbalance the cardiovascular benefits and hinder the clinical translation of full LXR agonists. Current drug development efforts aim to dissociate beneficial cholesterol-handling and anti-inflammatory LXR effects from lipogenic transcriptional programs. Approaches include selective LXR modulators (SLiMs) [112], isoform-specific ligands [113,114], and tissue-biased strategies exploiting subtle conformational differences between LXRα and LXRβ, differential cofactor recruitment, or restricted tissue expression to preserve cardioprotective effects while avoiding hepatic lipid accumulation. Resolving this balance is essential for realizing LXRs as viable therapeutic targets in atherosclerosis [115,116], MAFLD [117], metabolic syndrome, and chronic inflammatory disorders [107].

### 5.2. Strategies for Safer LXR Targeting

A major challenge in advancing LXR-directed therapies toward clinical application is achieving robust atheroprotective, anti-inflammatory, and cholesterol-efflux-enhancing activity while avoiding lipogenic and steatogenic effects driven predominantly by hepatic LXRα activation [115,116]. To overcome this barrier, multiple pharmacological strategies have been explored, including the development of isoform-selective ligands and tissue-targeted delivery platforms restricting LXR engagement to macrophages or the intestine. Another promising direction involves combining LXR agonists with inhibitors of downstream lipogenic pathway (ACC inhibitors, FASN inhibitors, SREBP-1c modulators) [118] to preserve LXR-mediated cholesterol efflux and anti-inflammatory signaling while suppressing hepatic triglyceride synthesis. Additional combinatorial strategies pair LXR activators with FXR or PPAR agonists to counterbalance sterol and lipid metabolic fluxes, thereby minimizing steatosis and enhancing overall therapeutic safety profiles of LXR-based interventions [118].

#### 5.2.1. Full LXRα/LXRβ Agonists

Full LXR agonists activate both LXRα and LXRβ with high efficacy, stabilizing canonical active receptor conformations (including helix 12 closure), resulting in maximal LXR target gene activation. While these ligands strongly induce cholesterol efflux pathways (upregulating *ABCA1*, *ABCG1*, *ApoE*), they simultaneously trigger SREBP-1c lipogenic programs, markedly increasing hepatic fatty acid and triglyceride synthesis. This dual action confers potent anti-atherogenic and anti-inflammatory properties but also leads to hepatic steatosis and hypertriglyceridemia, limiting translational potential.

**Figure 5:**
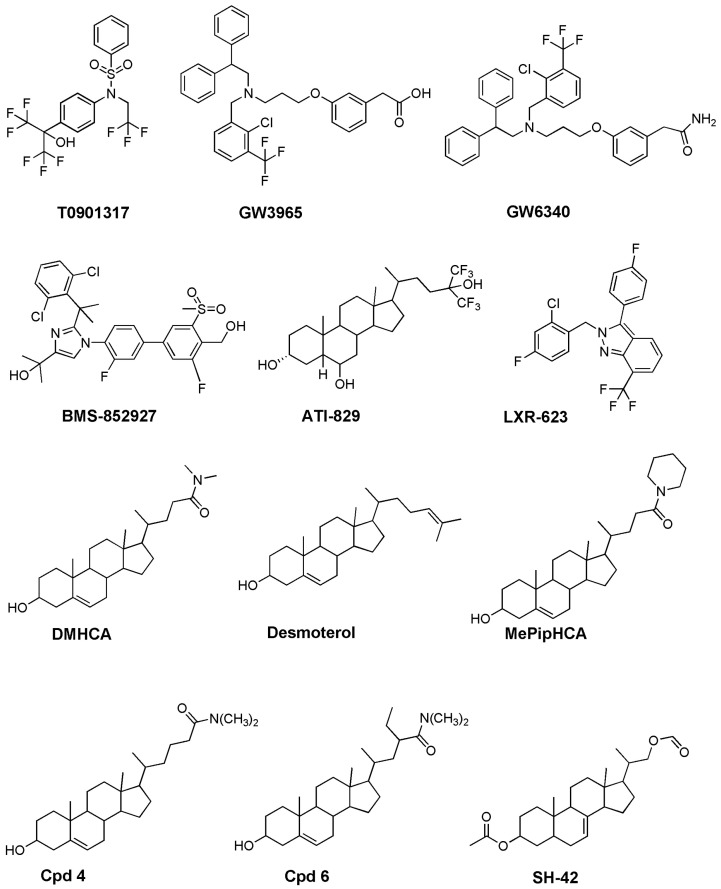
Chemical structures of LXR modulators.

**T0901317** (Figure 5), the first widely used synthetic LXR agonist, belongs to the diaryl-amino-acetate steroid mimetic class. Its structure includes a rigid diaryl core, tert-butyl and chloro substituents enhancing hydrophobic interactions with the LXR ligand-binding pocket, and a tertiary amide-containing side chain that stabilizes receptor-active conformations. **T0901317** is a highly potent agonist (EC_50_ approximately 20–50 nM for LXRα and LXRβ) and was pivotal in demonstrating LXR activation reduces cholesterol accumulation and suppresses inflammatory gene expression in macrophages [119]. However, it also robustly induces hepatic *SREBP-1c*, *FASN*, and stearoyl-CoA desaturase 1 (*SCD1*), producing pronounced lipogenesis, hepatic steatosis, and hypertriglyceridemia [119].

**GW3965** (Figure 5), another widely used full agonist, is an aryl-oxy-propionamide derivative characterized by a benzamide scaffold with dimethylamino substituents [120]. Developed as a more selective and pharmacokinetically favorable LXR ligand than **T0901317**, **GW3965** retains high potency and fully activates both LXR isoforms [120]. In animal models, **GW3965** increases high-density lipoprotein (HDL) levels, enhances RCT, and reduces atherosclerosis, yet it still strongly activates hepatic *SREBF1* (SREBP-1c*) *and lipogenesis [121], leading to hepatic triglyceride accumulation similar to **T0901317** (Table 5). Collectively, **T0901317** and **GW3965** represent first-generation, high-efficacy pan-agonists, revealing both the therapeutic promise of LXR activation and the major metabolic liabilities of full-strength LXRα stimulation. These limitations directly motivated the development of SLiMs, partial agonists, desmosterol-mimetic sterols, and tissue-restricted delivery strategies designed to uncouple cholesterol-efflux benefits from undesirable hepatic lipogenesis.

#### 5.2.2. LXRβ-Selective Agonists

Because LXRβ mediates anti-inflammatory and cholesterol-efflux pathways with limited hepatic lipogenesis induction, LXRβ-preferring agonists represent an attractive strategy for safer LXR modulation. Molecules such as **LXR-623 **(**WAY-252623**),** BMS-852927**, and the sterol-based partial agonist **ATI-829** have demonstrated reduced lipogenic potential relative to nonselective agonists like T0901317 [115,122].

**LXR-623** (2-[(2-chloro-4-fluorophenyl)methyl]-3-(4-fluorophenyl)-7-(trifluoromethyl)indazole) (Figure 5) [123] enhances cholesterol efflux and protects against atherosclerosis by activating LXR-regulated genes (*ABCA1*) while exhibiting minimal hepatic lipid metabolism effects. Its potency is strongly biased toward LXRβ, with EC_50_ values of approximately 100 nM for LXRβ and 1000–2000 nM for LXRα, reflecting greater than 10-fold selectivity. **LXR-623** was the first LXR agonist tested in humans. In a single-ascending-dose clinical study, it was rapidly absorbed (C_max_ approximately 2 h), displayed dose-proportional pharmacokinetics, and had a terminal half-life of 41–43 h. LXR activation resulted in dose-dependent *ABCA1* and *ABCG1* expression increases, and pharmacokinetic/pharmacodynamic (PK/PD) modeling estimated EC_50_ values of 526 ng/mL for *ABCA1* and 729 ng/mL for *ABCG1* [123]. Central nervous system (CNS)-related adverse effects emerged at the highest doses, but no deaths or serious adverse events occurred [123] (Table 5). This study represented the first demonstration of target engagement by an LXR agonist in humans [123].

**BMS-852927** (Figure 5) is a selective LXRβ partial agonist developed to stimulate RCT and reduce atherosclerosis while minimizing adverse lipid effects typically associated with full LXR agonists. In transactivation assays, it shows 20% LXRα and 88% LXRβ activity relative to a full pan-agonist. **BMS-852927 (XL041)** is highly potent, with an EC_50_ of 9 nM and 26% activity in a human whole-blood endogenous target gene activation assay (WBA). This benzimidazole–carboxamidine displays similar binding affinity for LXRα and LXRβ (19 and 12 nM, respectively) [124]. In preclinical monkey studies, **BMS-852927** increased cholesterol efflux by up to 50% and raised HDL levels by approximately 20% without meaningful triglyceride or LDL increases. However, in a Phase 1 human study, single doses of 10–300 mg produced dose-dependent LDL cholesterol increases (up to 40%) and triglyceride increases (up to 60%), along with HDL reductions at the highest doses. Neutropenia was observed in 25–30% of participants. Mechanistic analyses suggest this neutropenia arises from LXR-mediated enhancement of neutrophil clearance and altered cholesterol handling in hematopoietic cells (Table 5) [124]. Although **BMS-852927** activated RCT markers in humans, its unanticipated lipid and immune side effects, absent in animal models, highlight challenges of safely targeting LXR signaling for cardiovascular therapy [124].

A similar pattern is seen with **ATI-829 **(3α,6α,24-trihydroxy-24,24-di(trifluoromethyl)-5β-cholane) [125], a synthetic oxysterol analog evaluated as a potential anti-atherosclerotic agent. In vitro, **ATI-829** upregulates *ABCA1* expression in THP-1 macrophage-like cells, selectively activates LXR, and induces minimal *SREBF1 (*SREBP-1c) expression in HepG2 hepatoma cells, indicating low lipogenic activity. In LDL receptor knockout (LDLR^−/−^) mice, oral administration (10 mg/kg/day for 2–12 weeks) significantly induces LXR target genes (*ABCA1*, *ABCG5*, *ABCG8*) in macrophages and intestine without increasing hepatic triglycerides. Unlike full LXR agonists, **ATI-829** does not cause hypertriglyceridemia and exhibits atheroprotective effects, reducing innominate artery lesion area by approximately 60% and aortic root lesions by approximately 27% [126]. These results support **ATI-829** as a promising agent enhancing cholesterol transport while minimizing adverse lipogenic responses.

Although achieving complete isoform selectivity remains difficult due to high LXRα and LXRβ LBD homology, LXRβ-biased ligands illustrate that promoting macrophage cholesterol efflux while limiting hepatic SREBP-1c activation is possible. Belorusova et al. [127] further showed that ligands such as **BMS-852927,** which promote ABCA1 expression (thus cholesterol efflux) without inducing triglyceride synthesis, tend to stabilize helix 3 of LXRα rather than helix 12, the latter being more strongly associated with lipogenic activation, according to hydrogen/deuterium exchange mass spectrometry (HDX-MS) analyses.

#### 5.2.3. Selective LXR Modulators (SLiMs)

Selective LXR Modulators (SLiMs) fine-tune LXR activity by stabilizing receptor conformations, preferentially activating beneficial pathways while avoiding harmful ones. They promote cholesterol efflux (*ABCA1*, *ABCG1*), suppress inflammatory genes via transrepression, and minimize hepatic lipogenesis (*SREBF1 *, *FASN*, *SCD1*), reducing steatosis and hypertriglyceridemia risks. Prototypical SLiMs include **DMHCA, MePipHCA,** modified **GW3965 **derivatives, and more recently developed compounds such as compound** 4** and compound **6** (Figure 5), which selectively activate efflux pathways without elevating hepatic triglycerides.

**DMHCA **(*N,N*-dimethyl-3β-hydroxy-cholenamide) is a selective LXR agonist stimulating cholesterol efflux without triggering triglyceride synthesis, unlike earlier LXR agonists [128,129,130]. Mechanistically, **DMHCA** acts by directly activating LXR and by inhibiting the final cholesterol synthesis step, leading to desmosterol accumulation, an endogenous LXR agonist. This dual action allows **DMHCA** to promote ABCA1-mediated cholesterol efflux while minimally activating SREBP1c, reducing adverse effects seen with previous LXR modulators [128,129,130].

In diabetic db/db mice, **DMHCA** restores retinal cholesterol homeostasis, slows diabetic retinopathy progression, reduces systemic inflammation, and corrects bone marrow dysfunction [129,130]. Diabetes typically reduces LXR expression and oxysterol levels, leading to retinal cholesterol accumulation. **DMHCA** reverses these effects by inhibiting cholesterol synthesis, enhancing LXR-driven cholesterol efflux, and increasing oxysterol-mediated cholesterol removal. Functionally, **DMHCA** improves retinal vascular structure and vision, restores membrane fluidity in human diabetic CD34^+^ reparative cells, lowers inflammatory cytokines in bone marrow, and increases beneficial reparative cell populations [131] (Table 5). It also normalizes hematopoietic stem and progenitor cell (HSPC) abnormalities, boosting erythroid and megakaryocyte progenitor formation. Single-cell RNA sequencing identifies early lineage bias in hematopoiesis and reveals a novel AP1-high stem cell population preferentially generating erythroid progenitors. **DMHCA** expands this population and enhances erythropoiesis while upregulating LXR target genes, immediate early genes, and pathways associated with protein synthesis, mammalian target of rapamycin (mTOR), hypoxia, and nuclear factor erythroid 2-related factor 2 (Nrf2) signaling. Its amphipathic structure may also directly affect membrane properties, contributing to a broad range of biological effects.

**MePipHCA** (methylpiperidinyl-3β-hydroxycholenamide), like DMHCA, mimics desmosterol’s structure. Both strongly induce LXR target genes involved in cholesterol efflux (*ABCA1*, *ABCG1*) in macrophages without activating lipogenic programs. In mice, **DMHCA** and **MePipHCA** upregulate *ABCA1* in elicited macrophages without inducing liver lipogenesis, indicating macrophage-selective profiles [132,133]. These “transrepression-selective” agonists are promising for reducing macrophage cholesterol, as in atherosclerosis, without the adverse hepatic triglyceride accumulation seen with non-selective LXR agonists.

In 2019, Li et al. [134] reported steroid-based LXR agonists, compounds **4** and **6,** designed to stimulate cholesterol efflux functions of LXRs while avoiding hepatic triglyceride induction, a major limitation of earlier agonists (T0901317, GW3965). These compounds selectively promote RCT, particularly in macrophages, while minimizing liver lipogenesis. In animal studies, compound **4** did not elevate hepatic triglycerides, demonstrating favorable metabolic profiles. Both compounds reduce macrophage foam cell formation, preventing cholesterol-laden macrophage accumulation, a key step in atherogenesis. Structural studies suggest efflux-selective agonists preferentially stabilize helix 3 of LXRα, promoting *ABCA1*-mediated cholesterol efflux, while traditional agonists also stabilize helix 12, driving lipogenesis. Compounds **4** and **6** appear to favor efflux-active conformations, avoiding lipogenic pathways, exemplifying biased LXR modulation [134].

#### 5.2.4. Tissue-Biased Activation and Targeted Drug-Delivery Approaches

Since hepatocellular LXRα activation is the primary driver of lipogenesis, spatially restricting LXR agonism provides an effective strategy to reduce adverse metabolic effects. Intestine-restricted agonists, such as **GW6340** (an intestine-specific prodrug of **GW3965**, a full LXR agonist) (Figure 5), selectively enhance HDL formation and macrophage RCT without inducing hepatic steatosis [135]. Compared with systemic **GW3965**, **GW6340** significantly increased macrophage-derived [^3^H]-cholesterol fecal excretion (approximately 52% over control), indicating enhanced RCT. Importantly, **GW6340** induced intestinal LXR target genes (*ABCA1*, *ABCG5/8*) without affecting liver gene expression or elevating hepatic triglycerides.

Similarly, macrophage-targeted agonists, such as nanocarrier-delivered **T0901317, GW3965**-loaded liposomes**,** and polymeric nanoparticles functionalized with macrophage-targeting ligands, achieve potent anti-inflammatory and pro-efflux effects without engaging the liver [136]. For example, poly(lactic-co-glycolic acid)-block-poly(ethylene glycol) (PLGA-b-PEG) nanoparticles loaded with **GW3965 **(**NP-LXR**) strongly activate LXR target genes and suppress inflammatory mediators in macrophages both in vitro and in vivo while avoiding lipogenic gene induction in hepatocytes.

Zhang et al. (2017) demonstrated that **GW3965** encapsulated in collagen IV-targeted polymer-lipid hybrid nanoparticles (Col IV-GW-NPs) preferentially delivered the agonist to atherosclerotic plaques, increasing *ABCA1/ABCG1* expression in plaque macrophages, reducing macrophage content in lesions, and, unlike free GW3965, without raising hepatic lipid biosynthesis or plasma triglycerides [136] (Table 5). Overall, nanoparticle- or liposome-based delivery of LXR agonists enables cell-specific targeting, particularly to plaque macrophages, promoting cholesterol efflux, reducing inflammation, and decreasing foam-cell formation while avoiding hepatocyte lipogenic programs. Because liver (hepatocellular) LXRα activation is the main unwanted lipogenesis source, such spatial LXR agonism restriction effectively minimizes metabolic side effects.

#### 5.2.5. Partial Agonism and Signal-Amplitude Bias

Partial LXR agonists selectively activate cholesterol efflux and anti-inflammatory pathways while avoiding lipogenic gene induction. These molecules engage LXR at submaximal receptor occupancy, sufficient to induce target genes (*ABCA1*, *ABCG1*), enhancing RCT, but remaining below the threshold required to activate SREBP-1c, the master regulator of fatty acid and triglyceride synthesis. This pharmacological strategy allows partial agonists to achieve beneficial LXR activation effects (cholesterol efflux, inflammatory gene transrepression) without promoting hepatic steatosis or hypertriglyceridemia, providing safer pharmacological profiles.

**DMHCA (N,N-dimethyl-3β-hydroxy-cholenamide)** (Figure 5) is a steroidal, partial or “dissociated” LXR agonist. Unlike classical full LXR agonists, **DMHCA** provides many beneficial LXR activation effects, such as enhanced cholesterol efflux and anti-atherogenic activity, without stimulating hepatic lipogenesis or causing triglyceride accumulation [128]. In apolipoprotein E knockout (*apoE^−/−^*) mice, chronic **DMHCA** treatment for 11 weeks drastically reduced atherosclerotic plaque formation. Importantly, these mice did not develop liver steatosis or hypertriglyceridemia, problems commonly arising with other LXR agonists. **DMHCA** raised mRNA levels of efflux and cholesterol-catabolism genes (*ABCA1*, *ABCG1*, *CYP7A1*) while not inducing *SREBF1 (*SREBP-1c) (the master lipogenic regulator) in the liver [137]. In macrophages and foam cells in vitro, **DMHCA** strongly induced *ABCA1* expression, promoting cholesterol efflux. In the retina, **DMHCA** reduced total cholesterol content (especially unesterified cholesterol) without raising systemic triglycerides or cholesterol. Interestingly, in this context, **DMHCA** appeared to work not only via LXR activation but also by partially inhibiting the final cholesterol biosynthesis step, thereby reducing retinal cholesterol production [129] (Table 5). More recently, in diabetic mouse models (db/db mice), **DMHCA** treatment corrected both retinal and bone marrow dysfunction, restored cholesterol homeostasis, reduced inflammation, and improved vascular and hematopoietic parameters, again without promoting hepatic lipid accumulation [130,138]. Thus, **DMHCA** presents favorable therapeutic indices, does not markedly raise hepatic triglycerides, and avoids hepatosteatosis, a major barrier to clinical development of full LXR agonists.

**ATI-829 **(3α,6α,24-trihydroxy-24,24-di(trifluoromethyl)-5β-cholane) (Figure 5) is a steroidal LXR agonist [126]. When tested in vitro, ATI-829 activates LXRα (and to a lesser extent LXRβ) in reporter assays, though it is relatively weaker than potent non-steroidal agonists such as **T0901317.** In macrophage cells (THP-1), **ATI-829** robustly induces *ABCA1*, a key cholesterol efflux gene [126]. In hepatocytes (HepG2 cells, liver-derived cells), **ATI-829** is a poor *SREBF1* (SREBP-1c) inducer, the master lipogenesis regulator, compared to non-steroidal agonists like [139] (Table 5).

In vivo (LDLR^−/−^ mice fed a Western diet), oral **ATI-829** selectively upregulates LXR target genes in the intestine and macrophages but not in the liver. Accordingly, it does not raise hepatic triglycerides or cause hypertriglyceridemia. In these mice, **ATI-829** reduces atherosclerotic lesion development (e.g., innominate artery) [126,139]. A validated liquid chromatography-tandem mass spectrometry (LC-MS/MS) method shows **ATI-829** is detectable in mouse plasma with good stability and linear quantification over a wide concentration range. After oral administration (10 mg/kg), **ATI-829** reaches measurable plasma concentrations with favorable half-lives, supporting its viability for in vivo use [125,140].

#### 5.2.6. Modulation of Endogenous Sterol Pathways

Endogenous oxysterols, including desmosterol and 24(S),25-epoxycholesterol, serve as natural LXR ligands and exhibit signaling bias toward cholesterol efflux and anti-inflammatory pathways rather than lipogenesis. These oxysterols preferentially induce expression of genes involved in RCT (*ABCA1*, *ABCG1*) and suppress pro-inflammatory mediators, offering finely tuned, homeostatic LXR activation modes. One promising approach to leverage this endogenous pathway is inhibition of 24-dehydrocholesterol reductase (DHCR24), the enzyme responsible for the final cholesterol biosynthesis step. DHCR24 inhibition leads to desmosterol accumulation, a potent, naturally occurring LXR agonist. By enhancing desmosterol levels, this strategy promotes LXR-mediated cholesterol efflux and anti-inflammatory effects in tissues (liver, macrophages) while minimizing SREBP-1c-driven lipogenic program activation.

Preclinical studies have shown DHCR24 inhibition or treatment with desmosterol mimetics improves hepatic lipid handling, reduces inflammation, and mitigates steatosis without triggering unwanted triglyceride synthesis increases commonly observed with synthetic, non-selective LXR agonists. Zhou et al. (2023) [114] showed that pharmacological DHCR24 inhibition (using compound **SH42**) (Figure 5) increases endogenous desmosterol, a potent LXR agonist, in liver and plasma. In a mouse model of diet-induced MAFLD/MASH (APOE*3-Leiden.*CETP*), SH42 reduced liver steatosis and inflammation [114] (Table 5). These beneficial effects depended strictly on LXRα, and importantly, SH42 did not cause hyperlipidemia [114].

Importantly, this approach maintains alignment with physiological regulatory networks, potentially offering safer and more targeted alternatives to full LXR agonists. By exploiting endogenous sterol pathways, researchers can achieve selective LXR activity modulation, enhancing cholesterol homeostasis and immunomodulation while avoiding metabolic side effects.

## 6. Integrated View and Comparative Analysis

**Figure 6:**
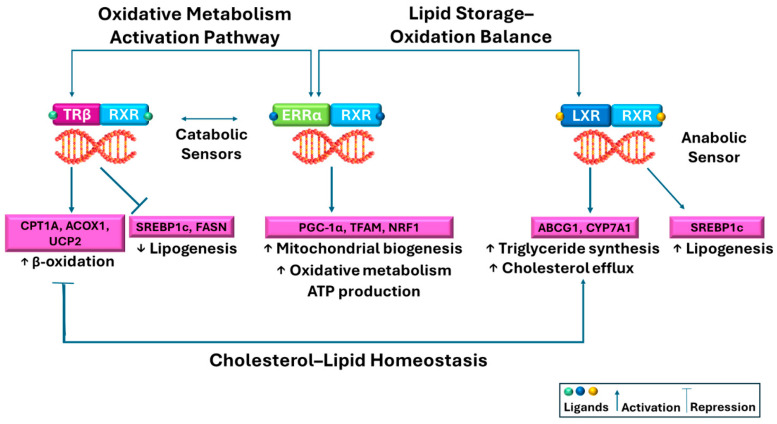
Convergent metabolic pathways in the liver: cross-talk among TRβ, ERRα, and LXR. Schematic representation illustrating cooperative and antagonistic interactions among TRβ, ERRα, and LXR in hepatic lipid metabolism. TRβ and ERRα drive catabolic programs (cholesterol clearance, FAO, mitochondrial biogenesis), while LXR promotes cholesterol efflux but also stimulates lipogenesis via SREBP-1c. These reciprocal interactions enable the liver to adaptively balance sterol protection, lipid storage, and oxidative energy production across diverse nutritional and endocrine states.

In the liver, NRs TRβ, ERRα, and LXR operate within a densely interconnected regulatory network integrating hormonal cues, nutrient status, and sterol load to fine-tune lipid and cholesterol homeostasis. These receptors share common structural domains, including highly conserved DBDs, and frequently bind similar direct repeat-4 (DR-4) type response elements as RXR heterodimers, creating intrinsic potential for cross-talk through cofactor competition and promiscuous recognition of AGGTCA half-sites [62,141]. TRβ and ERRα predominantly support catabolic programs: TRβ mediates TH-dependent induction of *LDLR* and *CYP7A1*, thereby accelerating cholesterol clearance and bile acid synthesis [142] while ERRα, largely through PGC-1α coactivation, drives mitochondrial biogenesis, OXPHOS, and fatty acid β-oxidation, particularly under fasting or high-energy-demand states [143]. In contrast, LXR functions primarily as an anabolic sterol sensor, activated by oxysterols to promote cholesterol efflux (*ABCA1*, *ABCG5/8*), conversion to bile acids, and robust induction of de novo lipogenesis through *SREBF1* [144]. This creates a multilayered interplay in which cooperation and antagonism coexist: TRβ and LXR jointly contribute to cholesterol disposal, although through partly distinct mechanisms, yet LXR-driven lipogenesis counteracts lipid-catabolic TRβ actions and oxidative ERRα programs [145]. ERRα further opposes LXR by repressing lipogenic genes while sustaining mitochondrial energy expenditure, thereby balancing LXR-mediated triglyceride synthesis during nutrient surplus. Collectively, these reciprocal interactions configure the liver as an adaptive metabolic hub capable of shifting between sterol protection, lipid storage, and oxidative energy production across feeding-fasting cycles and diverse endocrine states [143,144]. This integrated network underscores therapeutic potential of selectively targeting TRβ and LXR in dyslipidemia and CVD while highlighting species-specific differences, such as rodent-exclusive *CYP7A1* induction, that influence drug translation and efficacy. Recent clinical advances, including the FDA approval of **resmetirom** for MASH (March 2024), validate TRβ as a clinically actionable target and provide proof of concept for NR-based metabolic therapeutics [146]. Table 6 summarizes cross-talk among TRβ, ERRα, and LXR. Figure 6 highlights converging pathways, showing how cooperative and antagonistic interactions among TRβ, ERRα, and LXR influence hepatic lipid metabolism, cholesterol homeostasis, and overall energy balance.

## 7. Conclusions and Future Perspectives

The therapeutic landscape of metabolic disorders is undergoing a profound transformation driven by advances in nuclear receptor pharmacology. The expanding portfolio of modulators targeting TRβ, ERRα, and LXRα/β reflects a shift toward rational endocrine network control aimed at restoring metabolic homeostasis with increasing precision. Although these receptors regulate distinct yet interconnected biological pathways (TRβ governing hepatic lipid flux, ERRα coordinating mitochondrial energetics, and LXRs controlling cholesterol trafficking), their combined modulation exemplifies a broader transition from generalized metabolic interventions to targeted metabolic reprogramming.

Among these strategies, TRβ agonism currently represents the most clinically advanced approach. Liver-directed TRβ modulation has demonstrated robust efficacy in reducing hepatic steatosis, improving fibrosis, and normalizing systemic lipid profiles, establishing this pathway as a cornerstone for emerging therapies in metabolic liver disease. The recent clinical success of selective TRβ agonists provides clear proof of concept that tissue-selective nuclear receptor targeting can achieve therapeutic benefit while minimizing systemic endocrine liabilities. Continued optimization of liver-targeted delivery strategies, prodrug designs, and next-generation analogs is expected to further expand the therapeutic window of TRβ-based interventions.

ERRα, long considered challenging to target due to its constitutive activity and broad transcriptional reach, has recently emerged as a versatile metabolic regulator. Both agonists and inverse agonists have demonstrated beneficial effects depending on disease context, reflecting the receptor’s tissue-specific and pathway-dependent functions. Modulation of ERRα influences mitochondrial biogenesis, oxidative metabolism, lipid handling, and inflammatory signaling, highlighting its potential relevance for conditions ranging from insulin resistance and obesity to metabolic inflexibility and liver disease. These context-dependent effects position ERRα as a flexible pharmacological node rather than a unidirectional target.

LXRα/β modulation exemplifies the inherent complexity of nuclear receptor pharmacology. While activation of LXR pathways confers potent anti-atherogenic benefits through enhanced cholesterol efflux, excessive stimulation can provoke undesirable lipogenic responses in the liver. This duality has driven the development of selective, partial, biased, or tissue-restricted LXR modulators designed to decouple cardiovascular benefit from metabolic risk. Advances in structural biology and cofactor biology are increasingly enabling the rational design of ligands that favor cholesterol-handling programs while minimizing lipogenesis.

Across all three receptor classes, several shared challenges remain central to future progress. Achieving precise tissue selectivity is critical to avoid deleterious endocrine crosstalk, while the identification of robust, mechanism-based biomarkers is essential for patient stratification and therapeutic monitoring. Emerging insights into chromatin architecture, enhancer accessibility, and receptor cistromes underscore the importance of epigenomic context in shaping nuclear receptor responses and therapeutic outcomes. Integrating multi-omics approaches, advanced imaging, and transcriptional signatures will be instrumental in guiding precision endocrine therapies.

Looking ahead, the convergence of nuclear receptor biology with next-generation technologies promises transformative advances. Allosteric modulators, coregulator-biased ligands, combinatorial pharmacological strategies, and innovative modalities such as PROTACs and gene-regulatory tools offer unprecedented opportunities to fine-tune receptor activity with pathway- and tissue-level specificity. Beyond TRβ, ERRα, and LXRα/β, complementary modulation of other nuclear receptors, including glucocorticoid receptors and retinoid X receptors, may further enhance therapeutic precision by coordinating multiple metabolic axes simultaneously.

Collectively, these developments signal a decisive move toward precision endocrine modulation, in which therapeutic success is dictated by receptor selectivity, tissue context, transcriptional outcomes, and patient-specific metabolic architecture. Harnessing nuclear receptor signaling in this integrated manner holds the potential to transform the management of MAFLD, MASH, obesity, dyslipidemia, and related metabolic disorders, shifting clinical practice from broad metabolic manipulation to targeted, mechanism-driven metabolic rebalancing.

## Figures and Tables

**Table 1:** Global mortality attributable to key metabolic risk factors.

Risk Factor/Condition	Estimated Global Mortality/ Attributable Deaths	Key Sources
High LDL-Cholesterol (Hypercholesterolemia)	~4.40 million deaths in 2019 (3.78 million IHD, 0.61 million ischemic stroke)	[2,3]
High Fasting Plasma Glucose/Diabetes Risk	~6.50 million deaths in 2019, including cardiovascular, stroke, and kidney disease	[4,5,6]
High Body Mass Index (Obesity)	~5.02 million deaths in 2019, mostly cardiovascular	[7,8,9]
High Blood Pressure (Hypertension)	~10.85 million deaths in 2021	[9,10]
MASH	~34,700 deaths from liver cancer attributable to MASH	[11]

**Table 2:** Structural comparison of TRβ, ERRα, and LXR ligand-binding pockets.

Receptor	Ligand Type	PDB	Pocket Size/Shape	Polarity/Key Residues	Ligand-Pocket Interactions	Notes
TRβ	Agonist	1Q4X [41]	Small, elongated	S277, R320, H435; hydrophobic lining	H-bonds with polar residues; hydrophobic stacking	Highly selective; rigid pocket accommodates thyroid hormones
TRβ	Agonist	1R6G [42]	Small-medium, slightly expanded	M442 shifts to enlarge the cavity	Hydrophobic contacts; minimal H-bonds	Shows limited plasticity for bulkier ligands
ERRα	Inverse agonist	2PJL [44]	Large, reconfigured	F328, F510 H12 displaced	Hydrophobic; steric hindrance prevents coactivator binding	Classic inverse agonist mechanism
LXRα/β	Agonist	1UHL [48]	Wide, deep	H421, W443, L331	Van der Waals; contacts; H-bond	Accommodates diverse sterols; H12 in active conformation
LXRβ	Agonist	1P8D [49]	Wide, deep	H435, W457	H-bond “His–Trp switch”; hydrophobic contacts	Ligand-induced pocket plasticity
LXRβ	Agonist	1PQ6 [47]	Wide, deep	H435, H12, H3 and H7 residues	H-bond “His–Trp switch”; hydrophobic contacts	Ligand-induced pocket plasticity
LXRβ	Partial agonist	4DK8 [50]	Wide, adaptable	H3/H11/H12 residues	Hydrophobic reversed ligand orientation	Demonstrates flexibility for partial agonists

**Table 3:** TRβ compounds: selectivity, effects, and clinical progress.

Compound	TRβ:TRα Selectivity	Liver Targeting	Preclinical Effects	Clinical Effects	Status/Trials
**Sobetirome **(**GC-1**)	~10-fold	High liver accumulation	↓ hepatic steatosis, ↓ TG and cholesterol, ↓ AST/ALT; prevents lipid peroxidation	Phase 1: LDL-C ↓41%, safe	Phase 1 completed; discontinued (funding)
**GC-24**	40-fold	Moderate	↓ TG, ↓ fat mass, ↑ insulin sensitivity; limited cholesterol effect	None	Preclinical only
**Eprotirome **(**KB2115**)	High	High liver specificity	↓ hepatic steatosis, ↑ cholesterol secretion, ↓ LDL in rodents	↓ LDL ~40% in humans	Phase 3 terminated (cartilage toxicity)
**VK2809 **(**MB07811 → MB07344**)	High	Strong liver first-pass, minimal systemic exposure	↓ hepatic steatosis, ↓ TG and FFA, ↑ β-oxidation, ↑ mitochondrial respiration	↓ LDL-C and TG; liver fat ↓53–60% (MRI-PDFF); 52-week: MASH resolution, fibrosis regression	Phase 2b completed; ongoing development
**Resmetirom **(**MGL-3196/Rezdiffra™**)	28-fold	High liver specificity	↓ hepatic TG, ↓ steatosis, ↓ fibrosis and inflammation	Liver fat ↓33–37% (MRI-PDFF); ↓ LDL-C, ApoB, TG; MASH score improved (56%); FDA approved March 2024 (F2-F3 fibrosis)	FDA APPROVED
**IS25/TG68**	High	Liver-targeted	↓ hepatic lipid (HepG2), ↓ TG, ↓ liver weight, ↑ hepatocyte proliferation; no cardiac toxicity	None	Preclinical only
**SKL-12846/SKL-13784**	High	High liver specificity	↓ cholesterol, ↓ hepatic steatosis; minimal cardiac effects	None	Preclinical only
**Glucagon/T3 hybrid**	N/A	Liver + systemic metabolic targeting	↓ adipose mass, ↓ hepatic steatosis, ↓ atherosclerosis, ↑ glucose metabolism; reverses MASH; avoids thyrotoxicosis	None	Preclinical only

**Table 4:** ERRα modulators: preclinical development and therapeutic potential.

Compound	ERRα Selectivity/Type	Tissue Targeting	Preclinical Effects	Clinical Effects	Status/Trials
**XCT-790**	Inverse agonist	General/ Cellular	Downregulates ERRα transcription; off-target mitochondrial uncoupling	None	Preclinical only
**Compound** **29**	Selective antagonist	Liver, adipose, general metabolism	↓ body weight/fat mass, ↑ insulin sensitivity, ↓ glucose & lipid levels	None	Preclinical only (mouse/rat models)
**SLU-PP-332**	Pan-ERR agonist	Muscle, adipose, energy-demanding tissues	↑ mitochondrial gene expression & oxidative metabolism; ↑ energy expenditure; ↓ fat mass; ↑ insulin sensitivity	None	Preclinical only (mouse obesity/metabolic syndrome)
**SLU-PP-915**	Pan-ERR agonist	Muscle	↑ PGC1α, PDK4, LDHA; ↑ mitochondrial biogenesis and FAO; ↑ muscle endurance	None	Preclinical only (in vitro/mouse models)
**JND003**	ERRα-selective agonist	Liver, abdominal adipose	↑ hepatic oxidative metabolism; ↓ hepatic tTG; ↑ glucose tolerance and insulin sensitivity; ↓ liver ALT/AST	None	Preclinical only (HFD mice)
**“6c” (PROTAC-like degrader)**	ERRα degrader	Cellular	↓ ERRα protein by >80%; potential ↓ mitochondrial OXPHOS and FAO	None	Preclinical only (cellular studies)

**Table 5:** Preclinical and clinical development of LXR modulators, including full and partial agonists, β-selective agonists, SLiMs, tissue-targeted agents, and endogenous sterol-pathway modulators.

Compound	LXR Type/ Selectivity	Tissue Targeting	Preclinical Effects	Clinical Effects	Status/Trials
**T0901317**	Full pan-LXR agonist (LXRα/LXRβ)	Systemic	↑ *ABCA1/ABCG1*, ↑ HDL, anti-inflammatory; strong induction of *SREBP-1c, FASN, SCD1* → steatosis + ↑ TG	None	Widely used tool compound; preclinical only
**GW3965**	Full pan-LXR agonist	Systemic	↑ HDL, ↑ RCT, ↓ atherosclerosis, lipogenic like T0901317	None	Preclinical; no clinical development
**LXR-623 (WAY-252623)**	LXRβ-selective agonist	CNS-penetrant; systemic	↑ *ABCA1/ABCG1*; anti-atherosclerotic; minimal hepatic lipogenesis	In humans: ↑ *ABCA1/ABCG1* dose-dependently; CNS adverse events at high dose	Phase 1 completed
**BMS-852927**	LXRβ-preferential partial agonist	Systemic	↑ cholesterol efflux, ↑ HDL; minimal lipogenesis in primates	In humans: ↑ LDL and TG, ↓ HDL at high doses; neutropenia (25–30%)	Phase 1 completed (development halted)
**ATI-829**	Steroidal partial agonist/LXRβ-biased	Macrophages and intestine > liver	↑ *ABCA1*; ↓ atherosclerotic lesions; no hepatic TG increase	None	Preclinical only
**DMHCA**	SLiM, partial agonist, desmosterol-mimetic	Macrophage-selective; retina; bone marrow	↑ *ABCA1/ABCG1*; ↓ inflammation; ↓ atherosclerosis; no lipogenesis; improves diabetic retina and HSC dysfunction	None	Preclinical (multiple disease models)
**MePipHCA**	SLiM, desmosterol-like	Macrophages	Strong efflux activation in macrophages; no liver SREBP-1c activation	None	Preclinical only
**Compound 4**	SLiM efflux-selective	Macrophages	↑ RCT, ↓ foam cells; no hepatic TG elevation	None	Preclinical only
**Compound 6**	SLiM efflux-selective	Macrophages	↑ cholesterol efflux (*ABCA1*); low lipogenic signature	None	Preclinical only
**GW6340**	Intestine-restricted prodrug of GW3965	Intestine-specific	↑ HDL, ↑ macrophage-RCT; ↑ *ABCA1/ABCG5/8* intestinal; no activation of hepatic LXRα	None	Preclinical only
**GW3965 nanoparticles (PLGA-PEG, liposomes)**	Full agonist with targeted delivery	Macrophages/ atherosclerotic plaque	↑ *ABCA1/ABCG1*, ↓ inflammation, ↓ macrophage content in plaque; no hepatic lipogenesis	None	Preclinical only
**T0901317 nanoparticles/liposomes**	Full agonist with targeted delivery	Macrophages	Anti-inflammatory, efflux-promoting; avoids liver activation	None	Preclinical only
**SH42** **(DHCR24** **inhibitor)**	Endogenous sterol pathway modulator → ↑ desmosterol (LXR-biased endogenous agonism)	Liver, macrophages	↓ steatosis and ↓ inflammation; restores lipid homeostasis; no hypertriglyceridemia	None	Preclinical (MASH models)
**Desmosterol** **(endogenous)**	Natural LXR ligand (biased toward efflux)	Systemic (physiological)	↑ *ABCA1/ABCG1*, anti-inflammatory; minimal lipogenesis	Physiological ligand	N/A

**Table 6:** Mechanistic cross-talk among TRβ, ERRα, and LXR in hepatic metabolic regulation.

Mechanistic Feature	TRβ ↔ LXR	TRβ ↔ ERRα	ERRα ↔ LXR	Key References
Shared DNA Response Elements	Both bind DR-4 elements as RXR heterodimers; partial overlap in gene targets	Overlapping recognition of AGGTCA half-sites via NR family homology	ERRα can bind motifs similar to NR HREs; functional overlap with DR-4–regulated networks	[141]
Cofactor Recruitment Patterns	Compete for RXR and shared coactivators/ corepressors (e.g., SRC family)	Both recruit PGC-1 family members under specific conditions; competition with other NRs possible	ERRα strongly depends on PGC-1α, which can be diverted by other NRs, including LXR	[143]
Gene transcription interference	LXR-induced SREBP-1c opposes TRβ-mediated lipid catabolism; TRβ can repress lipogenic genes indirectly	Generally complementary; both enhance oxidative metabolism, reducing competition	ERRα activity influences transcriptional networks related to hepatic lipogenesis; inhibition or knockdown of ERRα has been shown to reduce expression of key lipogenic genes such as *FASN* and *ACACA*, functionally opposing LXR-driven lipogenic programs under certain conditions	[98]
Reciprocal Modulation of Target Gene Networks	TRβ promotes cholesterol clearance (*LDLR*, *CYP7A1*), counterbalancing LXR-driven lipogenesis; LXR enhances cholesterol efflux	TRβ enhances mitochondrial efficiency via thyroid hormone signaling, complementing ERRα programs	ERRα promotes FA oxidation, functionally counteracting LXR-driven Triglyceride synthesis	[141,142,143]
Biological Outcomes of Cross-Talk	Balances cholesterol detoxification vs. lipid catabolism; prevents excessive steatosis or lipid depletion	Enhances mitochondrial oxidative capacity and energy expenditure	Maintains equilibrium between lipid storage (LXR) and oxidation (ERRα)	[142,143,145]

## Data Availability

No new data were created or analyzed in this study. Data sharing is not applicable to this article.

## Abbreviations

NR: nuclear receptor; MAFLD, metabolic dysfunction-associated fatty liver disease; MASH, metabolic dysfunction-associated steatohepatitis; TRβ, thyroid hormone receptor β; ERRα, estrogen-related receptor α; LXR, liver X receptor; T2D, type 2 diabetes; CVD, cardiovascular disease; HCC, hepatocellular carcinoma; RXR, retinoid X receptor; HRE, hormone response element; PPAR, peroxisome proliferator-activated receptor; FXR, farnesoid X receptor; PXR, pregnane X receptor; TR, thyroid receptor; ERR, estrogen-related receptor; FAO, fatty acid oxidation; SPPARM, selective PPAR modulator; GLP-1 RA, glucagon-like peptide-1 receptor agonist; SGLT2i, sodium–glucose cotransporter-2 inhibitor; LBD, ligand-binding domain; DBD, DNA-binding domain; AF, activation function; TH, thyroid hormone; NIS, sodium/iodide symporter; LDLR, low-density lipoprotein receptor; SREBP-1c, sterol regulatory element-binding protein-1c; TAG, triacylglycerol; VLDL, very-low-density lipoprotein; LDL-C, low-density lipoprotein cholesterol; HFD, high-fat diet; MRI-PDFF, magnetic resonance imaging-proton density fat fraction; ALT, alanine aminotransferase; ApoB, apolipoprotein B; AMPK, AMP-activated protein kinase; ACC, acetyl-CoA carboxylase; ETC, electron transport chain; TCA, tricarboxylic acid cycle; NRF, nuclear respiratory factor; GPAT4, glycerol-3-phosphate acyltransferase 4; MTTP, microsomal triglyceride transfer protein; Pla2g12b, phospholipase A2 group XIIB; ROS, reactive oxygen species; NF-κB, nuclear factor kappa B; PDR, proliferative diabetic retinopathy; VEGF, vascular endothelial growth factor; MCP-1/CCL2, monocyte chemoattractant protein-1/C-C motif chemokine ligand 2; OGTT, oral glucose tolerance test; ZDF, Zucker diabetic fatty; ITT, insulin tolerance testing; PDK4, pyruvate dehydrogenase kinase 4; LDHA, lactate dehydrogenase A; *DDIT4*, DNA damage inducible transcript 4; MCAD, medium-chain acyl-CoA dehydrogenase; ATP5β, ATP synthase subunit beta; AST, aspartate aminotransferase; CPT1A, carnitine palmitoyltransferase 1A; PROTAC, proteolysis-targeting chimera; ApoA-I, apolipoprotein A-I; AP-1, activator protein 1; SLiM, selective LXR modulator; SCD1, stearoyl-CoA desaturase 1; HDL, high-density lipoprotein; PK/PD, pharmacokinetic/pharmacodynamic; CNS, central nervous system; WBA, whole-blood endogenous target gene activation assay; HDX-MS, hydrogen/deuterium exchange mass spectrometry; PLGA-b-PEG, poly(lactic-co-glycolic acid)-block-poly(ethylene glycol); HSPC, hematopoietic stem and progenitor cell; mTOR, mammalian target of rapamycin; Nrf2, nuclear factor erythroid 2-related factor 2; LC-MS/MS, liquid chromatography-tandem mass spectrometry; DHCR24, 24-dehydrocholesterol reductase; DR-4, direct repeat-4; HNF4α, hepatocyte nuclear factor 4 alpha; FOXA2, forkhead box protein A2; ATAC-seq, assay for transposase-accessible chromatin with sequencing; CUT&RUN, cleavage under targets and release using nuclease; HiChIP, Hi-C combined with chromatin immunoprecipitation; PCSK9, proprotein convertase subtilisin/kexin type 9; CRISPRa, CRISPR activation; CRISPRi, CRISPR interference.

## References

[1] GBD 2023 Disease and Injury and Risk Factor Collaborators (2025). Burden of 375 diseases and injuries, risk-attributable burden of 88 risk factors, and healthy life expectancy in 204 countries and territories, including 660 subnational locations, 1990-2023: A systematic analysis for the Global Burden of Disease Study 2023. Lancet.

[2] Du H., Shi Q., Song P., Pan X.F., Yang X., Chen L., He Y., Zong G., Zhu Y., Su B. (2022). Global Burden Attributable to High Low-Density Lipoprotein-Cholesterol From 1990 to 2019. Front. Cardiovasc. Med..

[3] World Heart Federation. https://world-heart-federation.org/.

[4] International Diabetes Federation. https://idf.org/.

[5] Liang R., Feng X., Shi D., Yang M., Yu L., Liu W., Zhou M., Wang X., Qiu W., Fan L. (2022). The global burden of disease attributable to high fasting plasma glucose in 204 countries and territories, 1990–2019: An updated analysis for the Global Burden of Disease Study 2019. Diabetes/Metab. Res. Rev..

[6] American Heart Association Inc. http://www.heart.org/.

[7] (2020). GBD-NHLBI-JACC Global Burden of Cardiovascular Diseases Writing Group. Global Burden of Cardiovascular Diseases and Risk Factors, 1990–2019. J. Am. Coll. Cardiol..

[8] Yao L., Hou W., Zheng Y., Su G. (2025). Global, regional, and national burden of cardiovascular diseases attributable to high body mass index from 1990 to 2021. Front. Cardiovasc. Med..

[9] Statista Worldwide Number of Deaths by Risk Factor. https://www.statista.com/statistics/1169367/worldwide-number-deaths-risk-factor/.

[10] Statista Worldwide Leading Causes of Death. https://www.statista.com/statistics/1488587/leading-causes-of-death-worldwide-2021.

[11] Pang J., Chen K., Chen S., Chen X. (2022). Global burden of nonalcoholic steatohepatitis-related liver cancer, 1990–2019: A systematic analysis for the GBD 2019. Diabetol. Metab. Syndr..

[12] Younossi Z.M., Golabi P., Paik J.M., Henry A., Van Dongen C., Henry L. (2023). The global epidemiology of nonalcoholic fatty liver disease (NAFLD) and nonalcoholic steatohepatitis (NASH): A systematic review. Hepatology.

[13] Cardiovascular Diseases (CVDs). https://www.who.int/news-room/fact-sheets/detail/cardiovascular-diseases-(cvds).

[14] GBD 2019 Risk Factors Collaborators (2020). Global burden of 87 risk factors in 204 countries and territories, 1990–2019: A systematic analysis for the Global Burden of Disease Study 2019. Lancet.

[15] Abdulrazzak E., Razzak I.A., Noureddin M., Trivedi H.D. (2025). A Gastroenterologist’s Approach to Improving Metabolic Health in MASLD. J. Clin. Gastroenterol..

[16] Wilding J.P.H., Batterham R.L., Calanna S., Davies M., Van Gaal L.F., Lingvay I., McGowan B.M., Rosenstock J., Tran M.T., Wadden T.A. (2021). Once-Weekly Semaglutide in Adults with Overweight or Obesity. N. Engl. J. Med..

[17] Newsome P.N., Buchholtz K., Cusi K., Linder M., Okanoue T., Ratziu V., Sanyal A.J., Sejling A.-S., Harrison S.A. (2021). A Placebo-Controlled Trial of Subcutaneous Semaglutide in Nonalcoholic Steatohepatitis. N. Engl. J. Med..

[18] Chawla A., Repa J.J., Evans R.M., Mangelsdorf D.J. (2001). Nuclear receptors and lipid physiology: Opening the X-files. Science.

[19] Abdel-Rasol M.A., El-Sayed W.M. (2025). Nuclear receptors in metabolic, inflammatory, and oncologic diseases: Mechanisms, therapeutic advances, and future directions. Eur. J. Med. Res..

[20] Weikum E.R., Liu X., Ortlund E.A. (2018). The nuclear receptor superfamily: A structural perspective. Protein Sci..

[21] Francis G.A., Fayard E., Picard F., Auwerx J. (2003). Nuclear receptors and the control of metabolism. Annu. Rev. Physiol..

[22] Sar P. (2023). Nuclear receptor: Structure and function. Prog. Mol. Biol. Transl. Sci..

[23] Shan X., Li D., Yin H., Tao W., Zhou L., Gao Y., Xing C., Zhang C. (2025). Recent Insights on the Role of Nuclear Receptors in Alzheimer’s Disease: Mechanisms and Therapeutic Application. Int. J. Mol. Sci..

[24] Lavecchia A., Cerchia C. (2018). Selective PPARγ Modulators for Type 2 Diabetes Treatment: How Far Have We Come and What Does the Future Hold?. Future Med. Chem..

[25] De Filippis B., Linciano P., Ammazzalorso A., Di Giovanni C., Fantacuzzi M., Giampietro L., Laghezza A., Maccallini C., Tortorella P., Lavecchia A. (2015). Structural development studies of PPARs ligands based on tyrosine scaffold. Eur. J. Med. Chem..

[26] Ibrahim A.M., Shoman M.E., El-Dien R.T.M., Saber E.A., Abdelnaser M., Maher S.A., Hayallah A.M., El-Rehany M.A.-A., Abuo-Rahma G.E.-D.A. (2025). Design and synthesis of a novel quinoline thiazolidinedione hybrid as a potential antidiabetic PPARγ modulator. Sci. Rep..

[27] Li Y., Ks N., Byran G., Krishnamurthy P.T. (2022). Identification of Selective PPAR-γ Modulators by Combining Pharmacophore Modeling, Molecular Docking, and Adipogenesis Assay. Appl. Biochem. Biotechnol..

[28] Xia H., Dufour C.R., Giguere V. (2019). ERRα as a Bridge Between Transcription and Function: Role in Liver Metabolism and Disease. Front. Endocrinol..

[29] Li L.-M., Song Y., Shi Y.-Q., Sun L.-L. (2023). Thyroid Hormone Receptor-β Agonists in NAFLD Therapy: Possibilities and Challenges. J. Clin. Endocrinol. Metab..

[30] Kininis M., Kraus W.L. (2008). A Global View of Transcriptional Regulation by Nuclear Receptors: Gene Expression, Factor Localization, and DNA Sequence Analysis. Nucl. Recept. Signal..

[31] Khorasanizadeh S., Rastinejad F. (2001). Nuclear-receptor interactions on DNA-response elements. Trends Biochem. Sci..

[32] Mangelsdorf D.J., Thummel C., Beato M., Herrlich P., Schutz G., Umesono K., Blumberg B., Kastner P., Mark M., Chambon P. (1995). The nuclear receptor superfamily: The second decade. Cell.

[33] Danielsen M. (2001). Bioinformatics of nuclear receptors. Methods Mol. Biol..

[34] Helsen C., Kerkhofs S., Clinckemalie L., Spans L., Laurent M., Boonen S., Vanderschueren D., Claessens F. (2012). Structural basis for nuclear hormone receptor DNA binding. Mol. Cell. Endocrinol..

[35] Nagy L., Schwabe J.W.R. (2004). Mechanism of the nuclear receptor molecular switch. Trends Biochem. Sci..

[36] Huang P., Chandra V., Rastinejad F. (2010). Structural overview of the nuclear receptor superfamily: Insights into physiology and therapeutics. Annu. Rev. Physiol..

[37] Frigo D.E., Bondesson M., Williams C. (2021). Nuclear receptors: From molecular mechanisms to therapeutics. Essays Biochem..

[38] Jin P., Duan X., Huang Z., Dong Y., Zhu J., Guo H., Tian H., Zou C.-G., Xie K. (2025). Nuclear receptors in health and disease: Signaling pathways, biological functions and pharmaceutical interventions. Signal Transduct. Target. Ther..

[39] Talukdar P.D., Chatterji U. (2023). Transcriptional co-activators: Emerging roles in signaling pathways and potential therapeutic targets for diseases. Signal Transduct. Target. Ther..

[40] Bahl S., Seto E. (2020). Regulation of histone deacetylase activities and functions by phosphorylation and its physiological relevance. Cell. Mol. Life Sci..

[41] Borngraeber S., Budny M.J., Chiellini G., Cunha-Lima S.T., Togashi M., Webb P., Baxter J.D., Scanlan T.S., Fletterick R.J. (2003). Ligand selectivity by seeking hydrophobicity in thyroid hormone receptor. Proc. Natl. Acad. Sci. USA.

[42] Hangeland J.J., Doweyko A.M., Dejneka T., Friends T.J., Devasthale P., Mellström K., Sandberg J., Grynfarb M., Sack J.S., Einspahr H. (2004). Thyroid receptor ligands. Part 2: Thyromimetics with improved selectivity for the thyroid hormone receptor beta. Bioorganic Med. Chem. Lett..

[43] Karnati K.R., Wang Y., Du Y. (2020). Exploring the binding mode and thermodynamics of inverse agonists against estrogen-related receptor alpha. RSC Adv..

[44] Kallen J., Lattmann R., Beerli R., Blechschmidt A., Blommers M.J.J., Geiser M., Ottl J., Schlaeppi J.-M., Strauss A., Fournier B. (2007). Crystal Structure of Human Estrogen-related Receptor α in Complex with a Synthetic Inverse Agonist Reveals Its Novel Molecular Mechanism. J. Biol. Chem..

[45] Malini N., Rajesh H., Berwal P., Phukan S., Balaji V.N. (2008). Analysis of Crystal Structures of LXRβ in Relation to Plasticity of the Ligand-Binding Domain upon Ligand Binding. Chem. Biol. Drug Des..

[46] Hoerer S., Schmid A., Heckel A., Budzinski R.-M., Nar H. (2003). Crystal Structure of the Human Liver X Receptor β Ligand-binding Domain in Complex with a Synthetic Agonist. J. Mol. Biol..

[47] Färnegårdh M., Bonn T., Sun S., Ljunggren J., Ahola H., Wilhelmsson A., Gustafsson J., Carlquist M. (2003). The Three-dimensional Structure of the Liver X Receptor β Reveals a Flexible Ligand-binding Pocket That Can Accommodate Fundamentally Different Ligands. J. Biol. Chem..

[48] Svensson S., Östberg T., Jacobsson M., Norström C., Stefansson K., Hallén D., Johansson I.C., Zachrisson K., Ogg D., Jendeberg L. (2003). Crystal structure of the heterodimeric complex of LXR and RXR ligand-binding domains in a fully agonistic conformation. EMBO J..

[49] Williams S., Bledsoe R.K., Collins J.L., Boggs S., Lambert M.H., Miller A.B., Moore J., McKee D.D., Moore L., Nichols J. (2003). X-ray Crystal Structure of the Liver X Receptor β Ligand Binding Domain. J. Biol. Chem..

[50] Kopecky D.J., Jiao X.Y., Fisher B., McKendry S., Labelle M., Piper D.E., Coward P., Shiau A.K., Escaron P., Danao J. (2012). Discovery of a new binding mode for a series of liver X receptor agonists. Bioorganic Med. Chem. Lett..

[51] Rubio I.G., Medeiros-Neto G. (2009). Mutations of the thyroglobulin gene and its relevance to thyroid disorders. Curr. Opin. Endocrinol. Diabetes.

[52] Chiamolera M.I., Wondisford F.E. (2009). Thyrotropin-Releasing Hormone and the Thyroid Hormone Feedback Mechanism. Endocrinology.

[53] Cheng S.-Y., Leonard J.L., Davis P.J. (2010). Molecular Aspects of Thyroid Hormone Actions. Endocr. Rev..

[54] Brent G.A. (2012). Mechanisms of thyroid hormone action. J. Clin. Investig..

[55] Sinha R.A., Singh B.K., Yen P.M. (2014). Thyroid hormone regulation of hepatic lipid and carbohydrate metabolism. Trends Endocrinol. Metab..

[56] Mullur R., Liu Y.-Y., Brent G.A. (2014). Thyroid Hormone Regulation of Metabolism. Physiol. Rev..

[57] Tao Y., Gu H., Wu J., Sui J. (2014). Thyroid function is associated with non-alcoholic fatty liver disease in euthyroid subjects. Endocr. Res..

[58] Bilgin H., Pirgon Ö. (2014). Thyroid Function in Obese Children with Non-Alcoholic Fatty Liver Disease. J. Clin. Res. Pediatr. Endocrinol..

[59] Hu L., Gu Y., Liang J., Ning M., Yang J., Zhang Y., Qu H., Yang Y., Leng Y., Zhou B. (2023). Discovery of Highly Potent and Selective Thyroid Hormone Receptor β Agonists for the Treatment of Nonalcoholic Steatohepatitis. J. Med. Chem..

[60] Weitzel J.M., Iwen K.A. (2011). Coordination of mitochondrial biogenesis by thyroid hormone. Mol. Cell. Endocrinol..

[61] Gullberg H., Rudling M., Saltó C., Forrest D., Angelin B., Vennström B. (2002). Requirement for Thyroid Hormone Receptor β in T_3_ Regulation of Cholesterol Metabolism in Mice. Mol. Endocrinol..

[62] Yen P.M. (2001). Physiological and molecular basis of thyroid hormone action. Physiol. Rev..

[63] Saponaro F., Sestito S., Runfola M., Rapposelli S., Chiellini G. (2020). Selective Thyroid Hormone Receptor-Beta (TRβ) Agonists: New Perspectives for the Treatment of Metabolic and Neurodegenerative Disorders. Front. Med..

[64] Zhao M., Xie H., Shan H., Zheng Z., Li G., Li M., Hong L. (2022). Development of Thyroid Hormones and Synthetic Thyromimetics in Non-Alcoholic Fatty Liver Disease. Int. J. Mol. Sci..

[65] Gullberg H., Rudling M., Forrest D., Angelin B., Vennström B. (2000). Thyroid Hormone Receptor β-Deficient Mice Show Complete Loss of the Normal Cholesterol 7α-Hydroxylase (CYP7A) Response to Thyroid Hormone but Display Enhanced Resistance to Dietary Cholesterol. Mol. Endocrinol..

[66] Mariash C.N. (2010). Thyroid hormone actions: To Beta or not to Beta. Thyroid.

[67] Ledda-Columbano G.M., Perra A., Concas D., Cossu C., Molotzu F., Sartori C., Shinozuka H., Columbano A. (2013). Synergism between obesity and alcohol in increasing the risk of hepatocellular carcinoma: A prospective cohort study. Am. J. Epidemiol..

[68] Hassan M.M., Kaseb A., Li D., Patt Y.Z., Vauthey J.-N., Thomas M.B., Curley S.A., Spitz M.R., Sherman S.I., Abdalla E.K. (2009). Association between hypothyroidism and hepatocellular carcinoma: A case-control study in the United States. Hepatology.

[69] Ledda-Columbano G.M., Perra A., Concas D., Cossu C., Molotzu F., Sartori C., Shinozuka H., Columbano A. (2003). Different effects of the liver mitogens triiodo-thyronine and ciprofibrate on the development of rat hepatocellular carcinoma. Toxicol. Pathol..

[70] Chiellini G., Apriletti J.W., Al Yoshihara H., Baxter J.D., Ribeiro R.C., Scanlan T.S. (1998). A high-affinity subtype-selective agonist ligand for the thyroid hormone receptor. Chem. Biol..

[71] Perra A., Simbula G., Simbula M., Pibiri M., Kowalik M.A., Sulas P., Cocco M.T., Ledda-Columbano G.M., Columbano A. (2008). Thyroid hormone (T3) and TRβ agonist GC-1 inhibit/reverse nonalcoholic fatty liver in rats. FASEB J..

[72] Freitas F.R.S., Moriscot A.S., Jorgetti V., Soares A.G., Passarelli M., Scanlan T.S., Brent G.A., Bianco A.C., Gouveia C.H.A. (2003). Spared bone mass in rats treated with thyroid hormone receptor TRβ-selective compound GC-1. Am. J. Physiol. Metab..

[73] Martagón A.J., Lin J.Z., Cimini S.L., Webb P., Phillips K.J. (2015). The Amelioration of Hepatic Steatosis by Thyroid Hormone Receptor Agonists Is Insufficient to Restore Insulin Sensitivity in Ob/Ob Mice. PLoS ONE.

[74] Scanlan T.S. (2008). Sobetirome: A case history of bench-to-clinic drug discovery and development. Hear. Fail. Rev..

[75] Vatner D.F., Weismann D., Beddow S.A., Kumashiro N., Erion D.M., Liao X.-H., Grover G.J., Webb P., Phillips K.J., Weiss R.E. (2013). Thyroid hormone receptor-β agonists prevent hepatic steatosis in fat-fed rats but impair insulin sensitivity via discrete pathways. Am. J. Physiol. Metab..

[76] Ladenson P.W., Kristensen J.D., Ridgway E.C., Olsson A.G., Carlsson B., Klein I., Baxter J.D., Angelin B. (2010). Use of the Thyroid Hormone Analogue Eprotirome in Statin-Treated Dyslipidemia. N. Engl. J. Med..

[77] Sjouke B., Langslet G., Ceska R., Nicholls S.J., E Nissen S., Öhlander M., Ladenson P.W., Olsson A.G., Hovingh G.K., Kastelein J.J.P. (2014). Eprotirome in patients with familial hypercholesterolaemia (the AKKA trial): A randomised, double-blind, placebo-controlled phase 3 study. Lancet Diabetes Endocrinol..

[78] Lammel Lindemann J., Webb P. (2016). Sobetirome: The past, present and questions about the future. Expert. Opin. Ther. Targets.

[79] Erion M.D., Cable E.E., Ito B.R., Jiang H., Fujitaki J.M., Finn P.D., Zhang B.-H., Hou J., Boyer S.H., van Poelje P.D. (2007). Targeting thyroid hormone receptor-β agonists to the liver reduces cholesterol and triglycerides and improves the therapeutic index. Proc. Natl. Acad. Sci. USA.

[80] Cable E.E., Finn P.D., Stebbins J.W., Hou J., Ito B.R., van Poelje P.D., Linemeyer D.L., Erion M.D. (2009). Reduction of hepatic steatosis in rats and mice after treatment with a liver-targeted thyroid hormone receptor agonist. Hepatology.

[81] Zhou J., Waskowicz L.R., Lim A., Liao X.H., Lian B., Masamune H., Refetoff S., Tran B., Koeberl D.D., Yen P.M. (2019). A Liver-Specific Thyromimetic, VK2809, Decreases Hepatosteatosis in Glycogen Storage Disease Type Ia. Thyroid.

[82] Kelly M.J., Pietranico-Cole S., Larigan J.D., Haynes N.E., Reynolds C.H., Scott N., Vermeulen J., Dvorozniak M., Conde-Knape K., Huang K.S. (2014). Discovery of 2-[3,5-dichloro-4-(5-isopropyl-6-oxo-1,6-dihydropyridazin-3-yloxy)phenyl]-3,5-dioxo-2,3,4,5-tetrahydro [1,2,4]triazine-6-carbonitrile (MGL-3196), a Highly Selective Thyroid Hormone Receptor β agonist in clinical trials for the treatment of dyslipidemia. J. Med. Chem..

[83] Harrison S.A., Bashir M.R., Guy C.D., Zhou R., Moylan C.A., Frias J.P., Alkhouri N., Bansal M.B., Baum S., Neuschwander-Tetri B.A. (2019). Resmetirom (MGL-3196) for the treatment of non-alcoholic steatohepatitis: A multicentre, randomised, double-blind, placebo-controlled, phase 2 trial. Lancet.

[84] Harrison S.A., Bashir M., Moussa S.E., McCarty K., Frias J.P., Taub R., Alkhouri N. (2021). Effects of Resmetirom on Noninvasive Endpoints in a 36-Week Phase 2 Active Treatment Extension Study in Patients With NASH. Hepatol. Commun..

[85] Madrigal Therapeutics Madrigal Announces Positive Topline Results from the Pivotal Phase 3 MAESTRO-NASH Clinical Trial of Resmetirom for the Treatment of NASH and Liver Fibrosis. https://ir.madrigalpharma.com/news-releases/news-release-details/madrigal-announces-positive-topline-results-pivotal-phase-3.

[86] FDA Approves First Treatment for Patients with Liver Scarring Due to Fatty Liver Disease. https://www.fda.gov/news-events/press-announcements/fda-approves-first-treatment-patients-liver-scarring-due-fatty-liver-disease.

[87] Runfola M., Sestito S., Bellusci L., La Pietra V., D’Amore V.M., Kowalik M.A., Chiellini G., Gul S., Perra A., Columbano A. (2020). Design, synthesis and biological evaluation of novel TRβ selective agonists sustained by ADME-toxicity analysis. Eur. J. Med. Chem..

[88] Perra A., Kowalik M.A., Cabras L., Runfola M., Sestito S., Migliore C., Giordano S., Chiellini G., Rapposelli S., Columbano A. (2020). Potential role of two novel agonists of thyroid hormone receptor-β on liver regeneration. Cell Prolif..

[89] Takahashi N., Maeda K., Asano Y., Watanabe N. (2014). Synthesis and pharmacological characterization of 1-benzyl-4-aminoindole-based thyroid hormone receptor β agonists. Bioorg. Med. Chem..

[90] Takahashi N., Asano Y., Maeda K., Watanabe N. (2014). In Vivo Evaluation of 1-Benzyl-4-aminoindole-Based Thyroid Hormone Receptor β Agonists: Importance of Liver Selectivity in Drug Discovery. Biol. Pharm. Bull..

[91] Finan B., Clemmensen C., Zhu Z., Stemmer K., Gauthier K., Müller L., De Angelis M., Moreth K., Neff F., Perez-Tilve D. (2016). Chemical Hybridization of Glucagon and Thyroid Hormone Optimizes Therapeutic Impact for Metabolic Disease. Cell.

[92] Schreiber S.N., Emter R., Hock M.B., Knutti D., Cardenas J., Podvinec M., Oakeley E.J., Kralli A. (2004). The estrogen-related receptor alpha (ERRalpha) functions in PPARgamma coactivator 1alpha (PGC-1alpha)-induced mitochondrial biogenesis. Proc. Natl. Acad. Sci. USA.

[93] Rui L. (2014). Energy metabolism in the liver. Compr. Physiol..

[94] Moore D.D. (2012). Nuclear receptors reverse McGarry’s vicious cycle to insulin resistance. Cell Metab..

[95] Giguère V., Yang N., Segui P., Evans R.M. (1998). Identification of a new class of steroid hormone receptors. Nature.

[96] Giguère V. (2002). To ERR in the estrogen pathway. Trends Endocrinol. Metab..

[97] Huss J.M., Garbacz W.G., Xie W. (2015). Constitutive activities of estrogen-related receptors: Transcriptional regulation of metabolism by the ERR pathways in health and disease. Biochim. Biophys. Acta.

[98] Chen C.-Y., Li Y., Zeng N., He L., Zhang X., Tu T., Tang Q., Alba M., Mir S., Stiles E.X. (2021). Inhibition of Estrogen-Related Receptor α Blocks Liver Steatosis and Steatohepatitis and Attenuates Triglyceride Biosynthesis. Am. J. Pathol..

[99] Mao L., Peng L., Ren X., Chu Y., Nie T., Lin W., Zhao X., Libby A., Xu Y., Chang Y. (2022). Discovery of JND003 as a New Selective Estrogen-Related Receptor α Agonist Alleviating Nonalcoholic Fatty Liver Disease and Insulin Resistance. ACS Bio. Med. Chem. Au..

[100] Yang M., Liu Q., Huang T., Tan W., Qu L., Chen T., Pan H., Chen L., Liu J., Wong C.-W. (2020). Dysfunction of estrogen-related receptor alpha-dependent hepatic VLDL secretion contributes to sex disparity in NAFLD/NASH development. Theranostics.

[101] Eskiocak B., Ali A., White M.A. (2014). The Estrogen-Related Receptor α Inverse Agonist XCT 790 Is a Nanomolar Mitochondrial Uncoupler. Biochemistry.

[102] Abu El-Asrar A.M., Nawaz M.I., Ahmad A., Siddiquei M.M., Allegaert E., Gikandi P.W., De Hertogh G., Opdenakker G. (2025). A key role of the PGC-1α/ERR-α pathway in regulation of angiogenic factors in proliferative diabetic retinopathy. Front. Endocrinol..

[103] Patch R.J., Searle L.L., Kim A.J., De D., Zhu X., Askari H.B., O’Neill J.C., Abad M.C., Rentzeperis D., Liu J. (2011). Identification of diaryl ether-based ligands for estrogen-related receptor α as potential antidiabetic agents. J. Med. Chem..

[104] Billon C., Schoepke E., Avdagic A., Chatterjee A., Butler A.A., Elgendy B., Walker J.K., Burris T.P. (2023). A Synthetic ERR Agonist Alleviates Metabolic Syndrome. J. Pharmacol. Exp. Ther..

[105] Hampton C.S., Sitaula S., Billon C., Haynes K., Avdagic A., Wanninayake U., Adeyemi C.M., Chatterjee A., Griffett K., Banerjee S. (2023). Development and pharmacological evaluation of a new chemical series of potent pan-ERR agonists, identification of SLU-PP-915. Eur. J. Med. Chem..

[106] Peng L., Zhang Z., Lei C., Li S., Zhang Z., Ren X., Chang Y., Zhang Y., Xu Y., Ding K. (2019). Identification of New Small-Molecule Inducers of Estrogen-related Receptor α (ERRα) Degradation. ACS Med. Chem. Lett..

[107] Im S.-S., Osborne T.F. (2011). Liver X Receptors in Atherosclerosis and Inflammation. Circ. Res..

[108] Tontonoz P., Mangelsdorf D.J. (2003). Liver X Receptor Signaling Pathways in Cardiovascular Disease. Mol. Endocrinol..

[109] Repa J.J., Turley S.D., Lobaccaro J.-M.A., Medina J., Li L., Lustig K., Shan B., Heyman R.A., Dietschy J.M., Mangelsdorf D.J. (2000). Regulation of Absorption and ABC1-Mediated Efflux of Cholesterol by RXR Heterodimers. Science.

[110] Repa J.J., Liang G., Ou J., Bashmakov Y., Lobaccaro J.M., Shimomura I., Shan B., Brown M.S., Goldstein J.L., Mangelsdorf D.J. (2000). Regulation of mouse sterol regulatory element-binding protein-1c gene (SREBP-1c) by oxysterol receptors, LXRalpha and LXRbeta. Genes Dev..

[111] Grefhorst A., Parks E.J. (2009). Reduced insulin-mediated inhibition of VLDL secretion upon pharmacological activation of the liver X receptor in mice. J. Lipid Res..

[112] Viennois E., Mouzat K., Dufour J., Morel L., Lobaccaro J.-M., Baron S. (2012). Selective liver X receptor modulators (SLiMs): What use in human health?. Mol. Cell. Endocrinol..

[113] Griffett K., Burris T.P. (2023). Development of LXR inverse agonists to treat MAFLD, NASH, and other metabolic diseases. Front. Med..

[114] Zhou E., Ge X., Nakashima H., Li R., van der Zande H.J.P., Liu C., Li Z., Müller C., Bracher F., Mohammed Y. (2023). Inhibition of DHCR24 activates LXRα to ameliorate hepatic steatosis and inflammation. EMBO Mol. Med..

[115] Savla S.R., Prabhavalkar K.S., Bhatt L.K. (2022). Liver X receptor: A potential target in the treatment of atherosclerosis. Expert. Opin. Ther. Targets.

[116] Calkin A.C., Tontonoz P. (2010). Liver X Receptor Signaling Pathways and Atherosclerosis. Arter. Thromb. Vasc. Biol..

[117] Kim H., Park C., Kim T.H. (2023). Targeting Liver X Receptors for the Treatment of Non-Alcoholic Fatty Liver Disease. Cells.

[118] Wan X., Ma J., Bai H., Hu X., Ma Y., Zhao M., Liu J., Duan Z. (2025). Drug Advances in NAFLD: Individual and Combination Treatment Strategies of Natural Products and Small-Synthetic-Molecule Drugs. Biomolecules.

[119] Schultz J.R., Tu H., Luk A., Repa J.J., Medina J.C., Li L., Schwendner S., Wang S., Thoolen M., Mangelsdorf D.J. (2000). Role of LXRs in control of lipogenesis. Genes Dev..

[120] Wang M., Thomas J., Burris T.P., Schkeryantz J., Michael L.F. (2003). Molecular determinants of LXRalpha agonism. J. Mol. Graph. Model..

[121] Joseph S.B., McKilligin E., Pei L., Watson M.A., Collins A.R., Laffitte B.A., Chen M., Noh G., Goodman J., Hagger G.N. (2002). Synthetic LXR ligand inhibits the development of atherosclerosis in mice. Proc. Natl. Acad. Sci. USA.

[122] Zhang Y., Breevoort S.R., Angdisen J., Fu M., Schmidt D.R., Holmstrom S.R., Kliewer S.A., Mangelsdorf D.J., Schulman I.G. (2012). Liver LXRα expression is crucial for whole body cholesterol homeostasis and reverse cholesterol transport in mice. J. Clin. Investig..

[123] Katz A., Udata C., Ott E., Hickey L., Burczynski M.E., Burghart P., Vesterqvist O., Meng X. (2009). Safety, Pharmacokinetics, and Pharmacodynamics of Single Doses of LXR-623, a Novel Liver X-Receptor Agonist, in Healthy Participants. J. Clin. Pharmacol..

[124] Kirchgessner T.G., Sleph P., Ostrowski J., Lupisella J., Ryan C.S., Liu X., Fernando G., Grimm D., Shipkova P., Zhang R. (2016). Beneficial and Adverse Effects of an LXR Agonist on Human Lipid and Lipoprotein Metabolism and Circulating Neutrophils. Cell Metab..

[125] Guo J., Peng D., Dai Q., Liao S., Wright B.J., van Breemen R.B. (2010). Quantitative analysis of 3alpha,6alpha,24-trihydroxy-24,24-di(trifluoromethyl)-5beta-cholane, a potent synthetic steroidal liver X receptor agonist in plasma using liquid chromatography-tandem mass spectrometry. J. Chromatogr. B Analyt Technol. Biomed. Life Sci..

[126] Peng D., Hiipakka R.A., Dai Q., Guo J., Reardon C.A., Getz G.S., Liao S. (2008). Antiatherosclerotic effects of a novel synthetic tissue-selective steroidal liver X receptor agonist in low-density lipoprotein receptor-deficient mice. J. Pharmacol. Exp. Ther..

[127] Belorusova A.Y., Evertsson E., Hovdal D., Sandmark J., Bratt E., Maxvall I., Schulman I.G., Åkerblad P., Lindstedt E.-L. (2019). Structural analysis identifies an escape route from the adverse lipogenic effects of liver X receptor ligands. Commun. Biol..

[128] Kratzer A., Buchebner M., Pfeifer T., Becker T.M., Uray G., Miyazaki M., Miyazaki-Anzai S., Ebner B., Chandak P.G., Kadam R.S. (2009). Synthetic LXR agonist attenuates plaque formation in apoE^−/−^ mice without inducing liver steatosis and hypertriglyceridemia. J. Lipid Res..

[129] El-Darzi N., Astafev A., Mast N., Saadane A., Lam M., Pikuleva I.A. (2018). N,N-Dimethyl-3β-hydroxycholenamide Reduces Retinal Cholesterol via Partial Inhibition of Retinal Cholesterol Biosynthesis Rather Than its Liver X Receptor Transcriptional Activity. Front. Pharmacol..

[130] Vieira C.P., Fortmann S.D., Hossain M., Longhini A.L., Hammer S.S., Asare-Bediako B., Crossman D.K., Sielski M.S., Adu-Agyeiwaah Y., Dupont M. (2020). Selective LXR agonist DMHCA corrects retinal and bone marrow dysfunction in type 2 diabetes. J. Clin. Investig..

[131] Fadini G.P., De Kreutzenberg S., Agostini C., Boscaro E., Tiengo A., Dimmeler S., Avogaro A. (2009). Low CD34+ cell count and metabolic syndrome synergistically increase the risk of adverse outcomes. Atherosclerosis.

[132] Muse E.D., Yu S., Edillor C.R., Tao J., Spann N.J., Troutman T.D., Seidman J.S., Henke A., Roland J.T., Ozeki K.A. (2018). Cell-specific discrimination of desmosterol and desmosterol mimetics confers selective regulation of LXR and SREBP in macrophages. Proc. Natl. Acad. Sci. USA.

[133] Magida J.A., Evans R.M. (2018). Rational application of macrophage-specific LXR agonists avoids the pitfalls of SREBP-induced lipogenesis. Proc. Natl. Acad. Sci. USA.

[134] Li C., Chen H., Chen X., Li Y., Hua P., Wei J., Song C., Gu Q., Zhou H., Zhang J. (2019). Discovery of tissue selective liver X receptor agonists for the treatment of atherosclerosis without causing hepatic lipogenesis. Eur. J. Med. Chem..

[135] Yasuda T., Grillot D., Billheimer J.T., Briand F., Delerive P., Huet S., Rader D.J. (2010). Tissue-Specific Liver X Receptor Activation Promotes Macrophage Reverse Cholesterol Transport In Vivo. Arter. Thromb. Vasc. Biol..

[136] Zhang X.Q., Even-Or O., Xu X., van Rosmalen M., Lim L., Gadde S., Farokhzad O.C., Fisher E.A. (2015). Nanoparticles containing a liver X receptor agonist inhibit inflammation and atherosclerosis. Adv. Healthc. Mater..

[137] Quinet E.M., Savio D.A., Halpern A.R., Chen L., Miller C.P., Nambi P. (2004). Gene-selective modulation by a synthetic oxysterol ligand of the liver X receptor. J. Lipid Res..

[138] Cermenati G., Giatti S., Cavaletti G., Bianchi R., Maschi O., Pesaresi M., Abbiati F., Volonterio A., Saez E., Caruso D. (2010). Activation of the liver X receptor increases neuroactive steroid levels and protects from diabetes-induced peripheral neuropathy. J. Neurosci..

[139] Peng D., Hiipakka R.A., Xie J.T., Dai Q., Kokontis J.M., Reardon C.A., Getz G.S., Liao S. (2011). A novel potent synthetic steroidal liver X receptor agonist lowers plasma cholesterol and triglycerides and reduces atherosclerosis in LDLR^−/−^ mice. Br. J. Pharmacol..

[140] Komati R., Spadoni D., Zheng S., Sridhar J., Riley K.E., Wang G. (2017). Ligands of Therapeutic Utility for the Liver X Receptors. Molecules.

[141] Glass C.K. (1994). Differential recognition of target genes by nuclear receptor monomers, dimers, and heterodimers. Endocr. Rev..

[142] Berkenstam A., Färnegårdh M., Gustafsson J. (2004). Convergence of lipid homeostasis through liver X and thyroid hormone receptors. Mech. Ageing Dev..

[143] Tanida T. (2022). Molecular dynamics of estrogen-related receptors and their regulatory proteins: Roles in transcriptional control for endocrine and metabolic signaling. Anat. Sci. Int..

[144] Scholtes C., Giguère V. (2022). Transcriptional control of energy metabolism by nuclear receptors. Nat. Rev. Mol. Cell Biol..

[145] Jakobsson T., Treuter E., Gustafsson J.-Å., Steffensen K.R. (2012). Liver X receptor biology and pharmacology: New pathways, challenges and opportunities. Trends Pharmacol. Sci..

[146] Bhushan S., Sohal A., Noureddin M., Kowdley K.V. (2025). Resmetirom: The first approved therapy for treating metabolic dysfunction associated steatohepatitis. Expert. Opin. Pharmacother..

